# Coordination environment tuning of nickel sites by oxyanions to optimize methanol electro-oxidation activity

**DOI:** 10.1038/s41467-022-30670-4

**Published:** 2022-05-25

**Authors:** Shanlin Li, Ruguang Ma, Jingcong Hu, Zichuang Li, Lijia Liu, Xunlu Wang, Yue Lu, George E. Sterbinsky, Shuhu Liu, Lei Zheng, Jie Liu, Danmin Liu, Jiacheng Wang

**Affiliations:** 1 The State Key Laboratory of High Performance Ceramics and Superfine Microstructure, Shanghai Institute of Ceramics, Chinese Academy of Sciences, Shanghai, 200050 China; 2 Center of Materials Science and Optoelectronics Engineering, University of Chinese Academy of Sciences, Beijing, 100049 China; 3 Beijing Key Laboratory of Microstructure and Properties of Solids, Faculty of Materials and Manufacturing, Beijing University of Technology, Beijing, 100124 China; 4 School of Materials Science and Engineering, Suzhou University of Science and Technology, 99 Xuefu Road, Suzhou, 215011 China; 5 Department of Chemistry, Western University, 1151 Richmond Street, London, ON N6A5B7 Canada; 6 Advanced Photon Source, Argonne National Laboratory, Argonne, IL 60439 USA; 7 Institute of High Energy Physics, Chinese Academy of Sciences, Beijing, 100049 China; 8 Hebei Provincial Key Laboratory Nonmetallic Materials, College of Materials Science and Engineering, North China University of Science and Technology, Tangshan, 063210 China

**Keywords:** Green chemistry, Electrocatalysis, Electrocatalysis, Coordination chemistry

## Abstract

To achieve zero-carbon economy, advanced anode catalysts are desirable for hydrogen production and biomass upgrading powered by renewable energy. Ni-based non-precious electrocatalysts are considered as potential candidates because of intrinsic redox attributes, but in-depth understanding and rational design of Ni site coordination still remain challenging. Here, we perform anodic electrochemical oxidation of Ni-metalloids (NiP_x_, NiS_x_, and NiSe_x_) to in-situ construct different oxyanion-coordinated amorphous nickel oxyhydroxides (NiOOH-TO_x_), among which NiOOH-PO_x_ shows optimal local coordination environment and boosts electrocatalytic activity of Ni sites towards selective oxidation of methanol to formate. Experiments and theoretical results demonstrate that NiOOH-PO_x_ possesses improved adsorption of OH* and methanol, and favors the formation of CH_3_O* intermediates. The coordinated phosphate oxyanions effectively tailor the *d* band center of Ni sites and increases Ni-O covalency, promoting the catalytic activity. This study provides additional insights into modulation of active-center coordination environment via oxyanions for organic molecules transformation.

## Abstract (web summary)

Coordination environments around metal sites are important in electrocatalysis. Here, Ni metalloid oxidation produces oxyanion doped amorphous Ni-oxyhydroxides where substitution with phosphorus is found to boost methanol electrooxidation activity.

## Introduction

Methanol (CH_3_OH) is one of the most important liquid C1 resources with wide applications due to the huge renewable production capacity^1–5^. Methanol electrooxidation reaction (MOR) in the anode has been considered to be a vital half-reaction in various electrochemical devices, such as direct methanol fuel cells (DMFCs)^5,6^. Normally, MOR proceeds through the formation and adsorption of CO (CO_ad_) or formate intermediates. In the route with CO_ad_, strong adsorption of CO_ad_ could poison the active sites, especially on the platinum group metals (PGMs), thus causing the rapid decay of MOR current. In another route, weak adsorption of formate intermediates on the catalyst surface could release the formate ions in the alkaline without emission of greenhouse gas CO_2_. And the resultant formate is an important chemical substance widely used in textile, printing, and pharmacy industry^4^. MOR is recently considered a promising strategy to replace oxygen evolution reaction (OER), reducing the energy consumption for hydrogen generation and co-producing high-value-added chemicals. However, MOR generally suffers from sluggish kinetics due to the multi-electron transfer process^5,7^. Moreover, the state-of-art electrocatalysts for MOR are high-cost PGMs, thus limiting their wide applications^5^. Thus, it has great demand to develop highly active and cost-effective electrocatalysts for transforming methanol into value-added formate.

Recently, Ni-based electrocatalysts have been explored for MOR in the anode, such as bimetallic alloy^2,8–10^, metal aerogels^7^, chalcogenides^1,4^, oxides^11^, hydroxides^5,12,13^. However, the reaction mechanism of these Ni-based electrocatalysts still remains uncertain. Generally, Ni-based electrocatalysts in the anode could undergo surface reconstruction into nickel oxyhydroxide (NiOOH) species during the OER^14–19^. Related studies also reported that the residual or adsorbed anionic species in NiOOH could modulate the electronic structure of active sites and thus improve anodic OER activity^20–23^. Similarly, in the organic selective oxidation reaction, NiOOH is also considered a critical active species^3,12,24–26^, but the effects of chemical coordination of different anionic species have not been systematically studied^25,27,28^. Specially, to the best of our knowledge, rare attention has been paid to the impact of anionic doping towards the MOR. Hence, it is of great significance to figure out the anion-modulated mechanism during methanol selective conversion on NiOOH-based electrocatalysts.

**Fig. 1:**
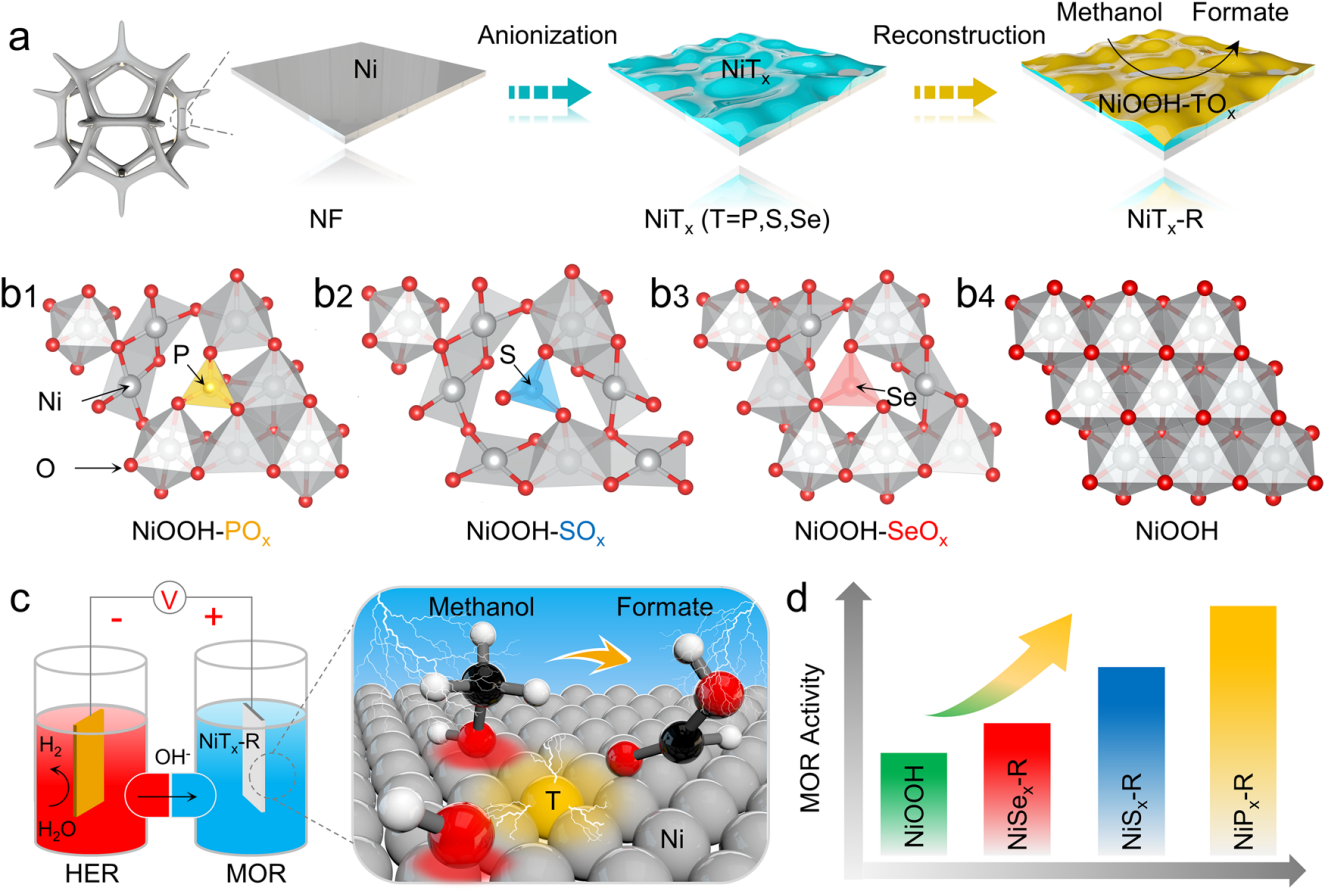
Synthesis of oxyanions-coordinated NiOOH through in situ surface reconfiguration towards MOR. **a** Schematic drawing of the synthesis route of NiT_x_-R electrocatalysts (T = P, S, and Se. And R means surface reconstruction) doped with different oxyanions on the surface NiOOH species (NiOOH-TO_x_) through surface anionization of nickel foams to prepare NiT_x_, followed by in situ electrochemical oxidation in the alkaline. **b**1–**b**4 Optimized structural models of oxyanions-doped NiOOH and pure NiOOH. **c** Schematic illustration for an H-type electrolytic cell and the related transformation mechanism of MOR for the NiT_x_-R electrocatalyst. **d** MOR activity comparison of pure NiOOH, NiP_x_-R, NiS_x_-R, and NiSe_x_-R. The results imply that phosphate-doped NiOOH prepared through in situ surface reconstruction of NiP_x_ exhibits the best MOR activity.

Here, we studied the influence of different oxyanions (TO_x_: T = P, S, and Se) on the coordination environments of Ni sites to optimize the electrocatalytic MOR performance. The Ni-metalloids (NiT_x_, T = P, S, and Se) were firstly prepared via surface anionization of nickel foams. Subsequently, active amorphous NiOOH coordinated with residual oxyanions (NiOOH-TO_x_) were constructed by in situ anodic electrochemical oxidation (Fig. 1a), leading to the formation of different coordination environments of Ni sites (Fig. 1b1–b3). Based on the various in situ and ex situ experiments, we confirm that the optimized local coordination environment of NiOOH with oxyanions can effectively regulate the adsorption of OH* intermediates and methanol molecules, and thus improve the MOR activity. Among the different samples, the NiOOH-PO_x_ derived from NiP_x_-R exhibits the best MOR activity (Fig. 1c, d). And the relationship between coordination environment and MOR activity is proposed by combining experimental characterizations with density functional theory (DFT) calculations.

## Results

### Preparation and structural characterizations of NiT_x_-R

**Fig. 2:**
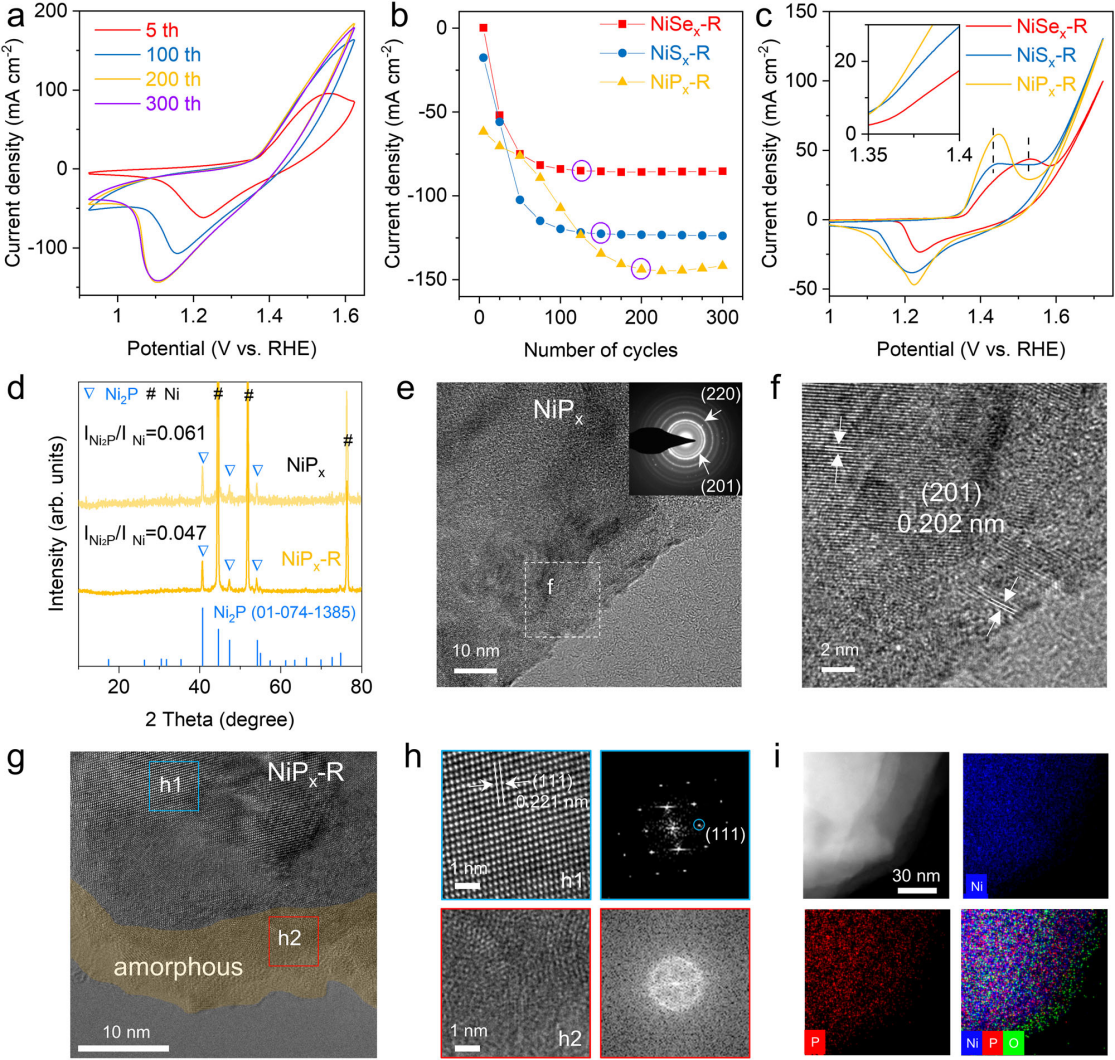
Activation preparation, structure, and morphology characterizations of NiP_x_-R. **a** Evolution of CV curves for NiP_x_ from the 5th to the 300th cycle in 1 M KOH at 100 mV s^−1^. **b** Cathodic peak current (I_pc_) as a function of cycle number. **c** CV curves for NiP_x_-R, NiS_x_-R, and NiSe_x_-R in 1 M KOH at 100 mV s^−1^. **d** XRD patterns for NiP_x_ and NiP_x_-R. **e** TEM image with SAED pattern (inset), and **f** corresponding HRTEM image of NiP_x_. **g**, **h** HRTEM images and the corresponding FFT patterns of the selected regions marked by the blue and red squares in (**g**). **i** HADDF-STEM image and corresponding elemental mappings of NiP_x_-R.

Ni-based oxyhydroxides coordinated with oxyanions (NiOOH-TO_x,_ T = P, S, and Se) were formed by anionization and in situ surface reconstruction^20^, as illustrated in Fig. 2a. First, Ni-metalloid (NiT_x_) alloys were prepared by a facile and straightforward anionization of commercial nickel foam (See details in Methods). Optical photos and scanning electron microscopy (SEM) images indicate that the obtained NiT_x_ (Supplementary Figs. 1–6) still maintains the porous framework of the pristine nickel foam (Supplementary Fig. 7). And the coverage of NiT_x_ on the nickel foam is uniform. Then, surface reconstruction was carried out on NiT_x_ by applying cyclic voltammetry (CV) in 1 M KOH medium at 100 mV s^−1^ from 0.924 to 1.624 V vs. reversible hydrogen electrode (RHE) for 300 cycles without iR compensation. As shown in Fig. 2a and Supplementary Fig. 8, the area of Ni oxidation peak (Ni^2+^ to Ni^3+^) and the OER current increase with the cycling numbers. Furthermore, the cathodic peak current (I_pc_) at different cycles was extracted to manifest the evolution (Fig. 2b). All NiT_x_ samples share a commonality that the reduction current gradually increases and tends to balance. It is noted that NiP_x_ needs more CV cycles (~200) than NiSe_x_ (~125) and NiS_x_ (~150) to achieve a stable current density. This suggests that NiP_x_-R has undergone a deeper reconstruction. To investigate the redox chemistry of the obtained NiT_x_-R, CV test with a scan rate of 5 mV s^−1^ was applied by using a typical three-electrode system in 1 M KOH. As shown in Fig. 2c, NiP_x_-R and NiS_x_-R show larger current densities than NiSe_x_-R. Notably, the onset potential of Ni oxidation peak for NiSe_x_-R is higher than those for NiP_x_-R and NiS_x_-R, indicating that a higher potential is needed to generate active specie (Ni^3+^)^29^. The NiP_x_-R shows the highest peak and area, mainly related to the large amount of redox-active Ni atoms^30,31^.

The detailed structural information was evaluated by X-ray diffraction (XRD) and high-resolution transmission electron microscopy (HRTEM). As shown in Fig. 2d, all the peaks can be well assigned to Ni foam substrate and Ni_2_P (PDF: 01-074-1385), which confirms the high crystallinity of as-obtained NiP_x_. After reconstruction, the signals of Ni and Ni_2_P still exist, but the peak intensity of Ni_2_P decreases slightly. Similarly, the peak intensity reduction is also found in both NiS_x_ and NiSe_x_ (Supplementary Fig. 9). The decrease in peak intensity derives from the partial dissolution of NiT_x_ during the reconstruction process, because inductively-coupled plasma-optical emission spectrometry (ICP-OES) confirms the presence of P, S, or Se elements in the electrolyte (Supplementary Fig. 10). In addition, the energy-dispersive spectrum (EDS) results of NiT_x_ and NiT_x_-R also demonstrate the leaching of oxyanions (Supplementary Fig. 11). These results suggest that the partial phase transformation and depletion of the NiT_x_ surface took place during surface reconstruction.

The HRTEM images of NiP_x_ (Fig. 2e, f and Supplementary Fig. 12) present lattice fringes with a spacing of 0.202 nm, which is well in accordance with the (201) facet of Ni_2_P. The corresponding selected-area electron diffraction (SAED) pattern (inset in Fig. 2e) shows bright rings, matching well with (201) and (220) plane of Ni_2_P. The HRTEM image of NiP_x_-R shows a crystalline-amorphous interface (Fig. 2g), suggesting a thin amorphous Ni hydroxide layer on the surface of Ni_2_P. The enlarged images are shown in Fig. 2h, where the diffraction spots (blue square) and diffused spot (red square) in the corresponding selected-area fast Fourier transform (FFT) patterns further confirm the crystalline and amorphous characters, respectively. To further prove the core-shell structure, we provide the high-angle annular dark-field scanning transmission electron microscopy (HAADF-STEM) image and the corresponding elemental mapping. As shown in Fig. 2i, the Ni and P elements are homogeneously distributed throughout the whole nanoplates, whereas the O element presents in the surface layer. In addition, the EDS line scanning result displays that the concentration of O gradually increases from the center to the surface (Supplementary Fig. 13). These results suggest that the amorphous Ni hydroxide is successfully formed on the NiP_x_ surface. The morphology and composition of the as-synthesized NiS_x_, NiSe_x_, NiS_x_-R, and NiSe_x_-R are also characterized by TEM and EDS (Supplementary Figs. 14–17). After reconstruction, the surface of NiS_x_ and NiSe_x_ also transformed into the amorphous hydroxide layer.

### Electronic structures and chemical environments of NiT_x_ and NiT_x_-R

**Fig. 3:**
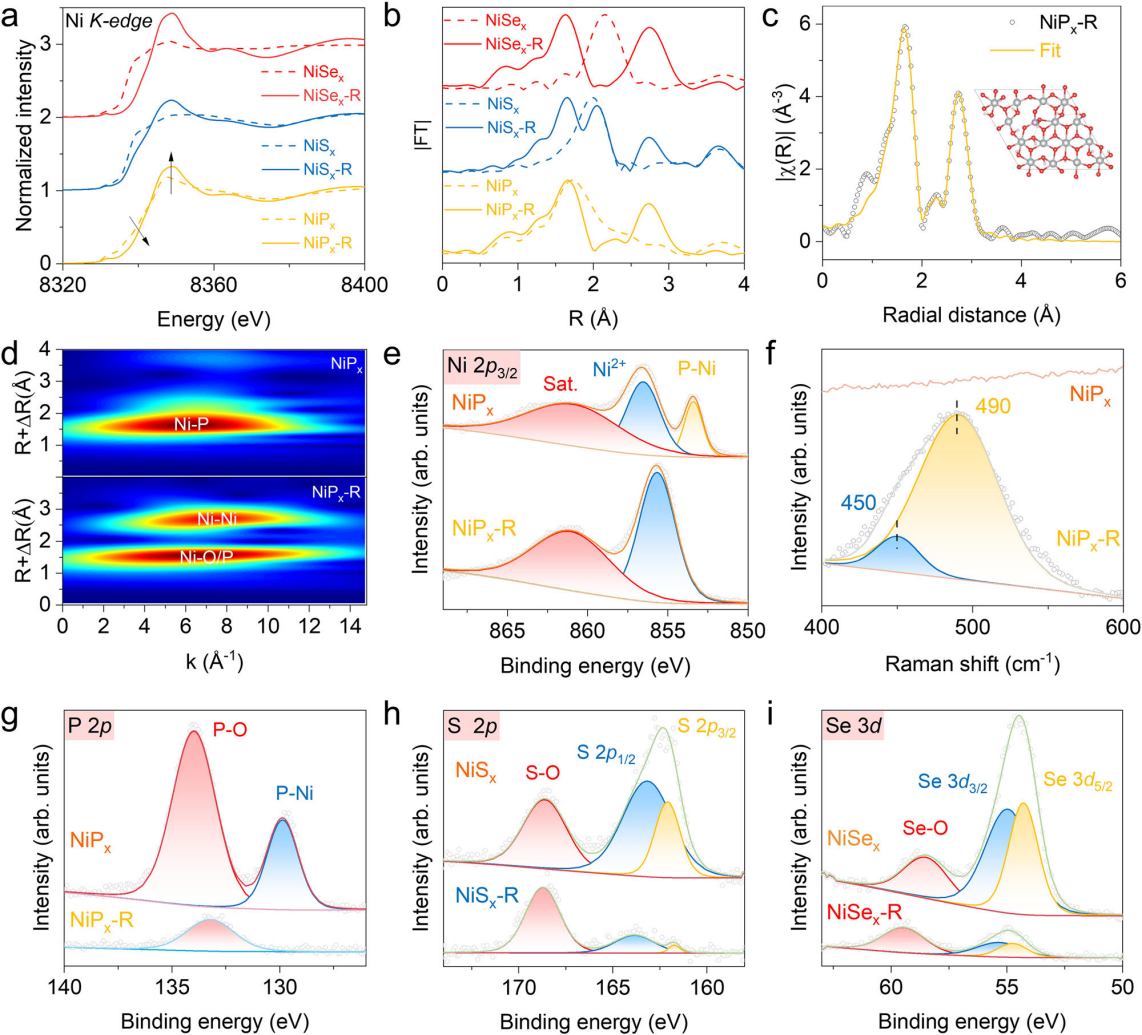
Electronic structures and chemical environments of NiT_x_ and NiT_x_-R. **a** XANES and **b** FT-EXAFS of Ni K-edge spectra for NiT_x_ and NiT_x_-R. **c** EXAFS fitting results of Ni K-edge at k-space of NiP_x_-R. **d** WT-EXAFS at Ni K-edge for NiP_x_ and NiP_x_-R. **e** High-resolution Ni 2*p* XPS spectra, **f** Raman spectra, and **g** high-resolution P 2*p* XPS spectra of NiP_x_ and NiP_x_-R. **h** High-resolution S 2*p* for NiS_x_ and NiS_x_-R. **i** High-resolution Se 3*d* for NiSe_x_ and NiSe_x_-R.

To further understand the transformation of NiT_x_ into NiT_x_-R by electrochemical oxidation, Ni K-edge X-ray absorption fine structure (XAFS) spectroscopy was conducted to track the changes in Ni local electronic structure and coordination environment. Figure 3a shows the Ni K-edge X-ray absorption near-edge structure (XANES) spectra of NiT_x_ and NiT_x_-R. The absorption thresholds of NiT_x_-R all occur at higher energy than those of their NiT_x_ counterparts at ~8340 eV, indicating the surface depletion of NiT_x_^1^.

The corresponding Fourier transform of extended X-ray absorption fine structure (FT-EXAFS) spectra is related to the radial distribution of neighboring atoms around the Ni atom. As shown in Fig. 3b, the FT-EXAFS spectra of NiP_x_ show a single peak around 1.8 Å at the initial state, which is assigned to Ni-P bonds in Ni_2_P. After reconstruction, the peak intensity of Ni-P bonds in NiP_x_-R decreases, and a new peak emerges at ~2.7 Å, which could be assigned to the Ni-Ni bonds in nickel hydroxide. This variation implies that the surface of NiP_x_ transformed to hydroxide, which is consistent with the observations of TEM. For NiS_x_ and NiSe_x_, similar structural evolution was also detected. To obtain quantitative binding information, EXAFS spectra were fitted with multiple scattering paths (Fig. 3c, Supplementary Fig. 18, and Supplementary Table 1). By utilizing the model in the inset of Fig. 3c, the simulated spectrum of backscattering signals χ^3^ (solid line) superimposes on the experimental one (dotted line), indicating a good match (Fig. 2c and Supplementary Fig. 18). According to the fitting results, it is rational to propose the coordination structure of NiP_x_-R is NiOOH-PO_x_. Other fitting processes for NiS_x_ and NiSe_x_ are similar to that of NiP_x_ (Supplementary Fig. 18). The Ni-O bond lengths and coordination numbers of three NiT_x_-R are found to be different from each other, which further verifies our proposed scheme for oxyanion function (Fig. 1). The wavelet transform of EXAFS spectra (WT-EXAFS) is powerful to distinguish the overlapped details in R-space by providing *k*-space resolution as well as radial distance resolution^32^. As shown in Fig. 3d and Supplementary Fig. 19, one intensity maximum at ~6.5 Å^–1^ in *k*-space assigned to Ni-P in NiP_x_ changes into two maxima at ~7.0 and 8.0 Å^–1^, which are ascribed to Ni-O/P and Ni-Ni, respectively. This variation further confirms the coordination change of Ni central atoms in NiP_x_-R compared with the fresh NiP_x_, which arises from the structural evolution of NiT_x_ to hydroxylation/oxidation during surface reconstruction.

The evolution of surface chemical states from NiT_x_ to NiT_x_-R was systematically studied by X-ray photoelectron spectroscopy (XPS). Figure 3e shows the high-resolution Ni 2*p* XPS spectra of NiP_x_ and NiP_x_-R. Compared with the fresh NiP_x_, the peak at ~853 eV assigned to metallic Ni (Ni-P) almost disappears in NiP_x_-R^33^. The Ni 2*p* spectra also display the binding energies of Ni 2*p*_3/2_ peaks located at 856 eV. These values are in good agreement with those of Ni^2+^ in nickel oxides or phosphates, which can be attributed to the surface oxidation of NiP_x_. After Ar ion etching, high-resolution XPS spectra of Ni 2*p* show that the Ni species exhibit stronger metallic character as the sputtering depth increases (Supplementary Fig. 20a). As for NiS_x_ and NiSe_x_, the oxidation of Ni can also be found after reconstruction (Supplementary Fig. 21). To further identify the structural difference between NiT_x_ and NiT_x_-R, Raman spectroscopy was employed (Fig. 3f). Compared to NiP_x_, there are two Raman peaks at 450 cm^−1^ and 490 cm^-1^ in the spectrum of NiP_x_-R, which can be assigned to the Ni-O vibration of Ni(OH)_2_ and the defective or disordered Ni(OH)_2_, respectively^34–36^. NiS_x_-R and NiSe_x_-R also demonstrate the similar features (Supplementary Fig. 22).

As for P 2*p* XPS spectra (Fig. 3g), the peaks at 130 and 134 eV belong to metal phosphide (Ni-P) and phosphate (P-O) species, respectively^37,38^. NiP_x_-R shows a stronger P-O peak than fresh NiP_x_. It is speculated that P in NiP_x_ is oxidized to phosphate. High-resolution XPS depth profiling spectra of P 2*p* show that as the sputtering depth increases, the P-O signal still exists, meaning the existence of PO_x_ in NiP_x_-R (Supplementary Fig. 20b). Compared with the fresh NiS_x_, NiS_x_-R demonstrates weaker peaks ascribed to metal-sulfur bindings (Ni-S) at 162 eV (S 2*p*_3/2_) and 163 eV (S 2*p*_1/2_), and a stronger S-O peak at ~168 eV (Fig. 3h), suggesting the surface oxidization during the CV activation^4,39^. Figure 3i shows the fitting peaks of Se 3*d* at 55 and 54 eV, agreeing well with the chemical states of Se 3*d*_3/2_ and Se 3*d*_5/2_. Besides, the peak at 59 eV is signified by the Se-O bond, indicating the surface is oxidized under an ambient condition^21,40^. After surface reconstruction, the atomic percentage of Se decreases from 73% to 10%, but the Se-O peak increases. As shown in Supplementary Fig. 23, the atomic percentage of different elements in NiT_x_ and NiT_x_-R were listed and compared, which clearly demonstrates the leaching and oxidation of T (T = P, S, or Se). The electronic configurations and local environments of oxyanions in NiP_x_-R and NiS_x_-R were also investigated by the P and S K-edge XANES (Supplementary Fig. 24), which imply the formation of new TO_x_ species with Ni sites in the surface-reconstructed samples. Additionally, electron paramagnetic resonance (EPR) spectroscopy shows that there are abundant oxygen vacancies (V_O_) in the amorphous nickel oxyhydroxides phase (Supplementary Fig. 25). Combining with all these characterization results, we can basically speculate that the surface composition of NiT_x_-R is a defect-rich amorphous nickel oxyhydroxides layer with residual oxyanions (TO_x_).

### Electrocatalytic MOR performance

**Fig. 4:**
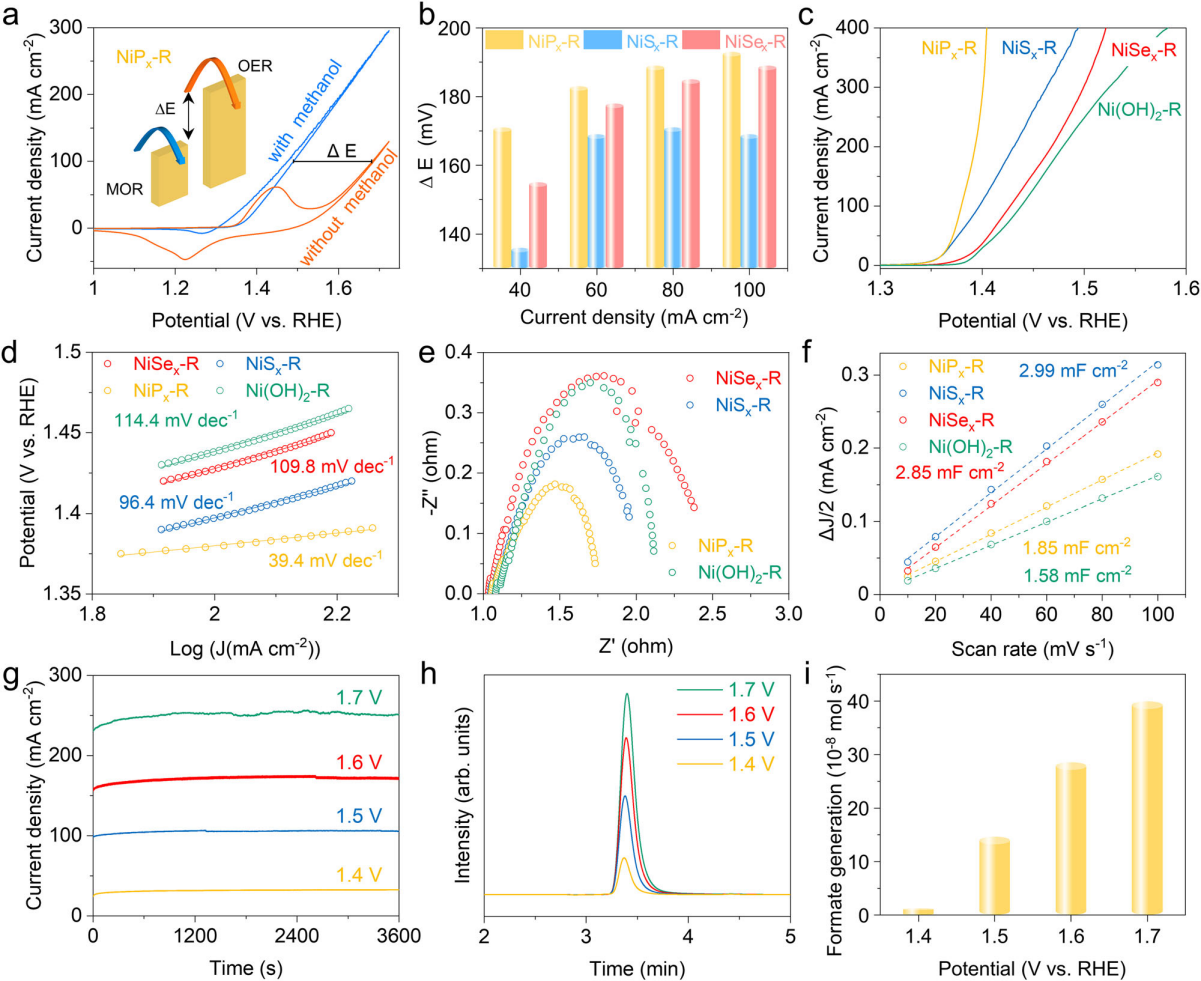
Electrocatalytic MOR performance. **a** CV curves of NiP_x_-R in 1 M KOH solution with and without 0.5 M methanol at a scan rate of 5 mV s^−1^. **b** The potential difference between MOR and OER at different current densities. **c** MOR polarization curves of NiT_x_-R and Ni(OH)_2_-R as the control sample. **d** Tafel plots, **e** electrochemical impedance spectra, and **f** the extracted double-layer capacitances (C_dl_) of different NiT_x_-R electrodes using a CV method. **g** Chronoamperometry (I–t) curves and **h** the IC traces of the diluted electrolyte for methanol upgrading reaction with the NiP_x_-R anode at 1.4–1.7 V (vs. RHE) for 1 h. **i** The averaged generation rates of formate at different potentials.

The electrochemical performances of the as-prepared electrodes were evaluated by an H-type electrolytic cell separated by an anion exchange membrane. As shown in Fig. 4a, NiP_x_-R requires a potential of 1.49 V to drive a current density of 100 mA cm^−2^ when catalyzing MOR in 1.0 M KOH with 0.5 M methanol, negatively shifting by 193 mV compared to that of OER. This result indicates that MOR can effectively decrease the overpotential of anode reaction (Fig. 4a, inset). As shown in Fig. 4b, compared with NiS_x_-R and NiSe_x_-R, NiP_x_-R shows the maximum reduction of potential between MOR and OER at different current densities (Supplementary Fig. 26). Linear sweep voltammograms (LSV) curves with iR compensation are shown in Fig. 4c. The potential of NiP_x_-R required to reach the current density of 400 mA cm^−2^ for MOR is 1.4 V, which is 90 and 117 mV lower than that of NiS_x_-R and NiSe_x_-R, respectively. It indicates that the Ni sites regulated by phosphate possess the best activity for MOR. Compared to reported Ni-based MOR catalysts (Supplementary Table 2), the obtained NiP_x_-R in this work exhibits low overpotential.

The superior reaction kinetics of NiP_x_-R for MOR is also verified by a much lower Tafel slope of 39.4 mV dec^−1^ than NiS_x_-R (96.4 mV dec^−1^) and NiSe_x_-R (109.8 mV dec^−1^) (Fig. 4d). Furthermore, the Nyquist plots (Fig. 4e) indicate that NiP_x_-R presents the lowest charge transfer resistance compared with NiS_x_-R and NiSe_x_-R. In addition, NiP_x_-R exhibits a much higher electrochemical surface area (ECSA) than other electrocatalysts, which is directly proportional to the double-layer capacitance (C_dl_) as shown in Fig. 4f and Supplementary Fig. 27. The ECSA-normalized LSV curves also show that NiP_x_-R exhibits the lowest overpotential to drive the same current density (Supplementary Fig. 27d), which indicates a high intrinsic activity.

The MOR by NiP_x_-R was also carried out by chronoamperometry (I–t) at different potentials for 1 h. The stable I–t curves indicate that the upgrading reactions are steady at 1.4–1.7 V vs. RHE (Fig. 4g). The identification and quantification of formate products were further conducted by Ion Chromatography (IC) based on the calibration curve (Fig. 4h). Figure 4i shows the generated formate concentrations by the NiP_x_-R anode at different potentials. The average generation rates of formate are 14.6 × 10^−8^, 28.5 × 10^−8^, and 39.9 × 10^−8^ mol s^−1^, at the working potential of 1.5, 1.6, and 1.7 V (vs. RHE), respectively.

To further verify the regulatory effect of oxyanions, we tested the XPS of NiP_x_-R after MOR. The XPS survey spectra display the signals of Ni, C, K, P, and O (Supplementary Fig. S28a). The Ni 2*p* spectra of NiP_x_-R after MOR (Supplementary Fig. 28b) show a pair of peaks at 855 (Ni 2*p*_3/2_) and 873 eV (Ni 2*p*_1/2_), which can be assigned to Ni^2+^. As shown in Supplementary Fig. S28c, the P 2*p* spectrum exhibits one peak at around 133 eV, which can be attributed to the P-O in PO_4_^3−^. This means the phosphate still exists after MOR. The C1 s spectrum shows an XPS peak at 288 eV (Supplementary Fig. S28d), which is ascribed to the functional group of O-C=O, probably owing to the residual formate species after MOR.

To exclude the effect of NF substrate and NiT_x_ thickness on the final MOR performance, we also prepared the NiT_x_ compounds with the same thickness on the surface of carbon paper (CP) (Supplementary Fig. S29–30). After the electrochemical reconstruction, their MOR performance was further evaluated. As shown in Supplementary Fig. S30, NiT_x_-R/CP samples also exhibit different MOR performance, and NiP_x_-R is still the best MOR catalyst. A detailed discussion can be seen in the Supplementary Information (Supplementary Figs. S29–31).

### MOR mechanism analysis

**Fig. 5:**
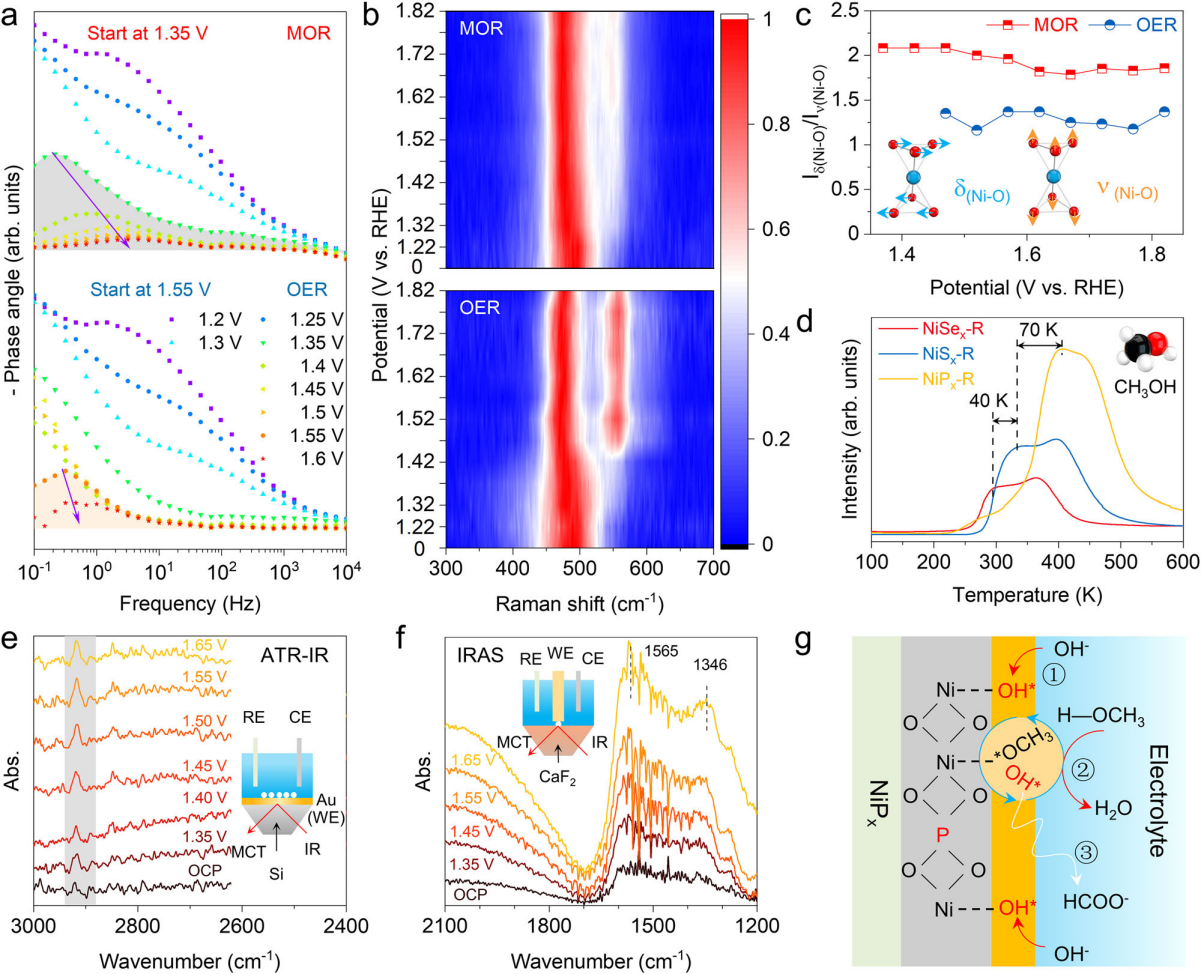
MOR mechanism analysis of NiP_x_-R by in situ and ex situ experiments. **a** Bode plots of NiP_x_-R for OER and MOR in different potentials. **b** In situ Raman spectroscopy of NiP_x_-R for OER (1 M KOH) and MOR (1 M KOH with 0.5 M methanol). **c** The δ_(Ni-O)_-to-ν_(Ni-O)_ ratios in the electrochemical in situ Raman spectra are related to the operating potentials. **d** Temperature-programmed desorption (TPD) curves of NiP_x_-R, NiS_x_-R, and NiSe_x_-R for methanol molecule. **e** ATR-IR spectra taken on the NiP_x_-R surface in the electrolyte of 0.1 M KOH with 0.5 M methanol in different potentials. **f** IRAS spectra of MOR on the NiP_x_-R surface in the electrolyte of 0.1 M KOH with 0.5 M methanol in different potentials. **g** Schematic illustration of the MOR mechanism on the NiP_x_-R surface.

To analyze the structure–activity relationship, the operando electrochemical impedance spectroscopy (EIS) was employed to probe the difference at different potentials during the OER or MOR process^24,41,42^. The Bode phase plots show that for NiP_x_-R, MOR occurs similar to OER at the low-frequency (0.1–10 Hz) interface (Fig. 5a), which proves that the MOR activity initiates from the same adsorption intermediate (OH*) as OER^41^. At low frequency, a transition peak can also be found at the potential of 1.35 and 1.55 V for NiP_x_-R during OER and MOR, which is consistent with their respective onset potential (Fig. 5a).

In situ Raman spectroscopy was carried out to further identify the low-frequency interface (Fig. 5b and Supplementary Fig. 32). In the Raman spectra, two peaks at 474 and 551 cm^−1^ correspond to Ni^3+^-O bending (δ(Ni-O)) and stretching (ν(Ni-O)), respectively^43^. After 1.42 V, the electrooxidation potential of NiP_x_-R, Ni^3+^-O of the oxide layer (Ni^2+^-O_x_H_y_) can be detected distinctly during OER. However, δ_(Ni-O)_ can be found after 1.32 V during MOR, it is suggested that Ni(OH)_2_ is oxidized into NiOOH and thus NiOOH would be the real active MOR catalyst. Notably, the variation of δ(Ni-O)-to-ν(Ni-O) ratio (labeled to I_δ/ν_) is obviously different for MOR and OER (Fig. 5c), signifying the distinguishing lattice structure of formed NiOOH. Generally, NiOOH contains β and γ phases, where the value of I_δ/ν_ in γ-NiOOH is higher due to its looser structure with more disorder^35,43^. As shown in Fig. 5c, the initial I_δ/ν_ is equal to 1.78 at the potential of 1.67 V during MOR, higher than 1.43 during OER. Such a higher I_δ/ν_ implies methanol molecules affect the local structure around Ni-O^35^.

Methanol molecule’s adsorption is the first step of MOR. The temperature-programmed desorption (TPD) measurements were performed to investigate the methanol adsorption behavior on NiT_x_-R (Fig. 5d)^44–46^. NiP_x_-R shows a higher desorption temperature (405 K) for methanol molecules than NiS_x_-R (335 K) and NiSe_x_-R (295 K), implying stronger methanol adsorption on NiP_x_-R. We speculate that the oxyanion-coordinated NiOOH promotes the adsorption and activation of the CH_3_OH molecule, thus resulting in superior MOR activity.

To further get insights into the MOR mechanism on NiP_x_-R, in situ infrared reflection spectra (IR) was carried out to monitor the intermediates and products. Firstly, we detected the characteristics of MOR on NiP_x_-R using in situ attenuated total reflection infrared spectroscopy (ATR-IR) (inset in Fig. 5e) in a 0.1 M KOH electrolyte with 0.5 M methanol. As shown in the ATR-IR spectra (Fig. 5e), the weakly adsorbed peaks at 2920 cm^−1^ increase slightly from 1.35 to 1.65 V, which is related to the surface CH_3_O* species generation^3^. Then, infrared reflection absorption spectroscopy (IRAS) was employed to monitor the products in electrolytes at each potential (inset in Fig. 5f). The broad peak from 1700 to 1200 cm^−1^, especially at 1565 and 1346 cm^−1^, is ascribed to the formation of formate^3,47^. Based on the above results, a basic understanding of the MOR mechanism on NiP_x_-R is gained (Fig. 5g): (1) the initial adsorption of OER intermediates (OH*) and CH_3_OH molecule at the surface of NiP_x_-R; (2) the reaction of OH* and CH_3_OH; (3) the oxidation of CH_3_O* and formation of HCOO^−^.

### DFT calculations

**Fig. 6:**
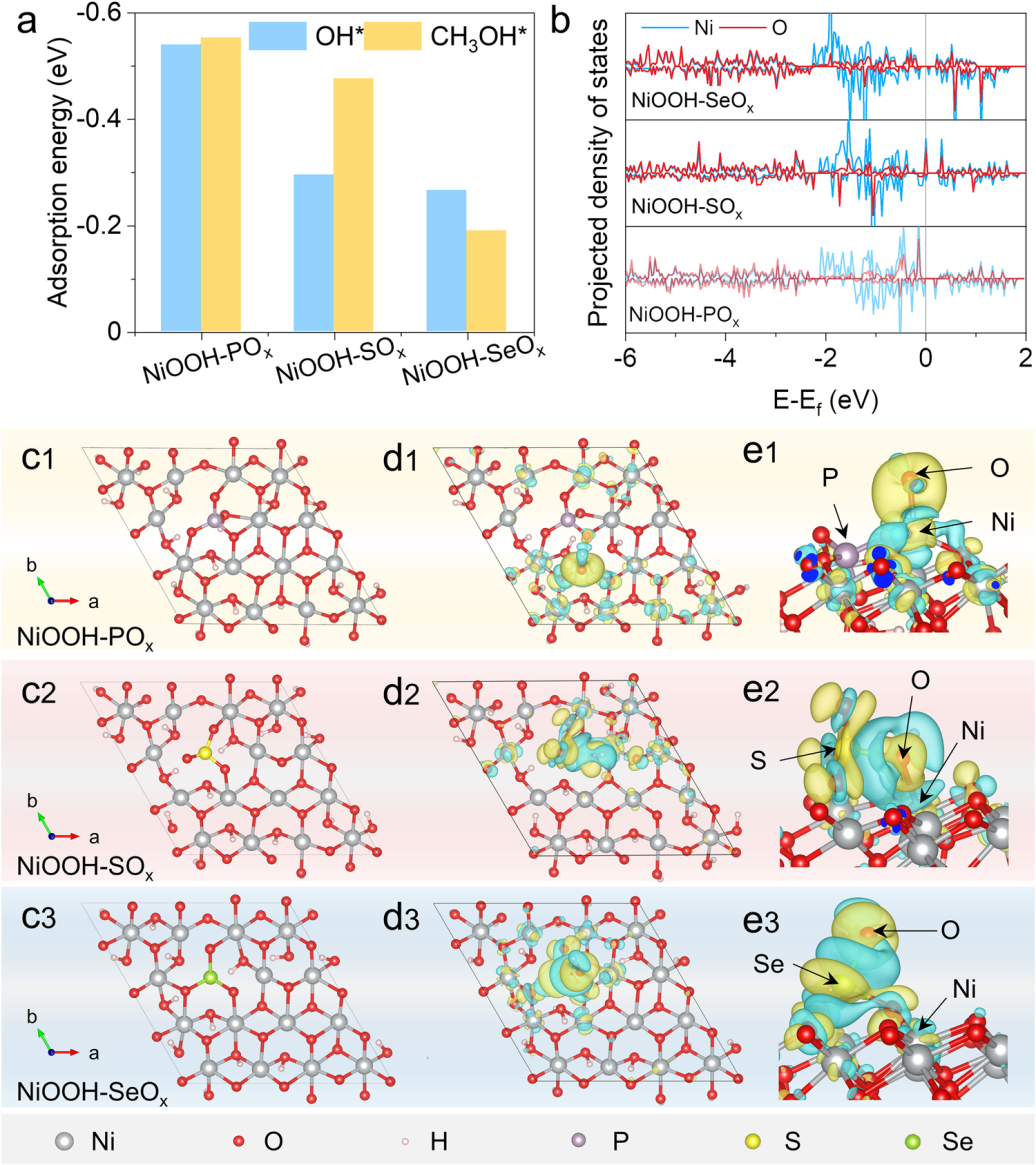
DFT calculations. **a** Adsorption energies of OH* and CH_3_OH* on NiOOH-PO_x_, NiOOH-SO_x_, and NiOOH-SeO_x_. **b** PDOS plots of Ni 3*d* and O 2*p* band for NiOOH-PO_x_, NiOOH-SO_x_, and NiOOH-SeO_x_. **c**1–**c**3 The slab models of NiOOH-PO_x_, NiOOH-SO_x_, and NiOOH-SeO_x_. **d**1–**d**3, **e**1–**e**3 Charge density difference of OH intermediates adsorption on NiOOH-PO_x_, NiOOH-SO_x_, and NiOOH-SeO_x_, respectively. Cyan and yellow represent the depletion and accumulation of electrons, respectively.

To elucidate the underlying reason for the activity difference of NiT_x_-R for MOR, density functional theory (DFT) calculations were performed by constructing NiOOH models with different oxyanions based on the surface reconstruction of NiT_x_-R. Firstly, the adsorption energy of OH* and CH_3_OH* on NiOOH-TO_x_ was calculated via DFT, respectively (Fig. 6a and Supplementary Fig. 33). NiOOH-PO_x_ shows stronger adsorption energy for both OH* and CH_3_OH* than NiOOH-SO_x_ and NiOOH-SeO_x_, which is beneficial for the initial adsorption and activation of CH_3_OH. The density of states (DOS) of the surface models in Fig. 1b1–b3 reveal that the Ni 3*d* band center (ε_*d*_) of NiOOH-PO_x_, NiOOH-SO_x_, and NiOOH-SeO_x_ is −1.68, −1.63, and −2.01 eV, respectively, suggesting that the ε_*d*_ could be effectively tailored by oxyanions (Fig. 6b). NiOOH-PO_x_ possesses the moderate adsorption of guest molecules, which conforms to the *d* band center theory and Sabatier principle^4,48^. Besides, the energy difference (ΔE) between ε_d_ and O 2*p* band center (ε_*p*_) of NiOOH-PO_x_, NiOOH-SO_x_, and NiOOH-SeO_x_ is calculated to be 3.3, 4.2, and 5.2 eV, respectively (Supplementary Table 3). This indicates that NiOOH-PO_x_ exhibits the strongest Ni 3*d*-O 2*p* orbital hybridization and the greatest Ni-O covalency among them. Previous studies have shown that a higher Ni-O covalency can promote the electron transfer between Ni atoms and oxygen adsorbates, thus accelerating OER process^22,49,50^. During the MOR process, the initial step is also the adsorption of OH^-^ species as revealed by in situ EIS characterization (Fig. 5a) and previous studies^1,4,41^. Therefore, the high Ni-O covalency tunes the OH binding energy during MOR processes.

As shown in Fig. 6c1–c3, the introduction of different oxyanions into NiOOH leads to the formation of unsaturated coordinated Ni. Finally, the charge density difference between before and after adsorption of OH* on the fivefold coordinated Ni site was also calculated, as shown in Fig. 6d1–d3 and 6e1–e3. The calculation results reveal the obvious electron transfer from the oxygen atoms of OH to Ni species in NiOOH-PO_x_ (Fig. 6d1, e1). However, for NiOOH-SO_x_, the electrons transfer from the oxygen atoms of OH* to both S and Ni sites (Fig. 6d2, e2) is unfavorable for the electron transport and successive reaction proceeding. As for NiOOH-SeO_x_ (Fig. 6d3, e3), the OH* directly binds with the Se atom after geometric optimization, demonstrating that the Ni sites at the surface of NiOOH-SeO_x_ have a very weak adsorbing ability for OH*, which is not favorable for the startup of MOR. Therefore, appropriate *d* band center and fluent charge transfer endow NiOOH-PO_x_ with superior MOR performance.

## Discussion

In summary, a series of oxyanions-doped amorphous Ni oxyhydroxide catalysts (NiOOH-TO_x_: T = P, S, or Se) were prepared by in situ surface reconstruction of Ni-metalloid (NiT_x_) through electrochemical activation. Among three kinds of oxyanions (TO_x_), the phosphate ions show the best ability to optimize the local coordination environment and boost the electrocatalytic activity of Ni sites towards selective oxidation of methanol to formate in the alkaline. The in situ and ex situ characterization experiments demonstrate that phosphate-coordinated NiOOH could enhance the adsorption of OH* and methanol, and promote the formation of CH_3_O* intermediates. Meanwhile, DFT calculations prove that the oxyanion coordination can effectively tailor the *d* band center of Ni sites. And the phosphate doping results in the highest Ni-O covalency, promoting the intermediate adsorption and catalytic activity. This work offers additional insights into understanding the effect of oxyanions on MOR performance and opens a fresh avenue for developing highly efficient electrocatalysts.

## Methods

### Chemicals

Nickel foam was purchased from Saibo Electrochemistry (Beijing, China). Sodium hypophosphite monohydrate (NaH_2_PO_2_•H_2_O), and potassium hydroxide (KOH) were purchased from Sinopharm Chemical Reagent Co., Ltd (Shanghai, China). Sulfur power (S) was purchased from Thermo Fisher Scientific. Methanol (CH_3_OH) and Selenium (Se) were purchased from Adamas. All the above chemicals were directly used without further purification.

### Synthesis of Ni-metalloid (NiT_x_: T = P, S, or Se)

To synthesize NiP_x_, a dried nickel foam (3 × 7 cm^2^) was put next to sodium hypophosphite monohydrate (NaH_2_PO_2_•H_2_O, 200 mg) under Ar atmosphere in the middle of a tube furnace. After flushing with Ar for ~60 min, the sample was heated to 400 ^o^C with a heating rate of 5 ^o^C min^−1^ for 1.5 h and then programmed to cool to ambient temperature.

To synthesize NiS_x_, a dried nickel foam (3 × 7 cm^2^) was put next to the sulfur power (S, 300 mg) under the Ar atmosphere in the middle of the tube furnace. After flushing with Ar for ~60 min, the sample was heated to 250 ^o^C with a ramping rate of 5 ^o^C min^−1^ under a flowing inert atmosphere for 0.5 h and then programmed to cool to ambient temperature.

To synthesize NiSe_x_, a dried nickel foam (3 × 7 cm^2^) was put next to the selenium powder (Se, 600 mg) under Ar atmosphere at the middle of the tube furnace. After flushing with Ar for ~60 min, the sample was heated to 350 ^o^C with a heating rate of 5 ^o^C min^−1^ for 2 h and then programmed to cool to ambient temperature.

### Synthesis of NiT_x_-R

NiT_x_-R was prepared by electrochemical activation of the NiT_x_ sample via applying cyclic voltammetry (CV) in 1 M KOH medium at 100 mV s^−1^ from 0.924 to 1.624 V vs. RHE for 300 cycles without iR compensation.

### Material characterization

The scanning electron microscopy (SEM) images were obtained using a field emission scanning electron microscope (FEI Magellan 400 L XHR). Transmission electron microscopy (TEM), high-resolution TEM (HRTEM), high-angle annular dark-field scanning TEM (HADDF-STEM), and energy-dispersive X-ray spectroscopy (EDS) mapping were taken on Titan G2 60-300 Cs-corrected TEM. X-ray diffraction (XRD) measurements were carried out on a Bruker D8 ADVANCE X-ray diffraction diffractometer. X-ray photoelectron spectroscopy (XPS) measurements were conducted with a Thermo ESCALAB250xi electron spectrometer using an Al Kα source (1486.6 eV) as a radiation source. Elemental analysis was conducted by an inductively-coupled plasma-optical emission spectrometry (ICP-OES) on the Agilent 5100. Electron Paramagnetic Resonance (EPR) spectra were acquired using a Bruker A300 spectrometer A300. Temperature-programmed desorption (TPD) experiments were conducted using AutoChem II 2920 (Micromeritics Instrument Corporation).

X-ray absorption fine structure (XAFS) spectra at the Ni K-edge were obtained at the Advanced Photon Source (APS), beamline 9-BM. The samples were pressed into pellets and measured in the X-ray fluorescence mode. The data were processed with the ATHENA program for background subtraction, normalization, and energy calibration^51^. The extended XAFS (EXAFS) was processed using the IFEFFIT package^52^. The EXAFS fitting was performed in R-space between 1.0 and 3.2 Å (the Fourier transform from k-space was performed over a range of 3.0 to 13.9 Å^−1^). Three scattering paths, which are Ni-O, Ni-T (T = S, P), and Ni-Ni, respectively, were used for the EXAFS fitting.

Phosphorus and sulfur K-edge X-ray absorption near-edge spectroscopy (XANES) measurements were performed on beamline 4B7A at the Beijing Synchrotron Radiation Facility (BSRF). The powder sample was spread evenly on the conductive double-sided carbon adhesive tape stuck on the sample holder. Spectra of the standard samples and NiP_x_ and NiS_x_ were recorded under total electron yield mode, while NiP_x_-R and NiS_x_-R were measured under partial fluorescence mode using silicon drifted detector (RaySpec, UK).

In situ Raman spectra were recorded on a micro-Raman spectrometer (Renishaw) under an excitation of 532 nm laser light under controlled potentials by the CHI 630E electrochemical workstation. The electrochemical operando Raman Cell was provided by the Beijing Scistar Technology Co., Ltd. In addition, the working electrode was covered by a catalyst. A Pt wire as the counter electrode was rolled to a circle around the cell. Ag/AgCl electrode (sat. KCl) was used as the reference electrode. The in situ Raman spectra were collected under chronoamperometry (I-t) at different potentials in a 1.0 M KOH solution.

In situ Fourier transformed infrared (FTIR) spectra were recorded on a Thermo Scientific™ Nicolet™ iS50 FTIR Spectrometer. The Attenuated Total Reflection (ATR) and Infrared Reflection Absorption Spectroscopy (IRAS) measurements were performed on a spectro-electrochemical cell provided by Linglu Instrument (Shanghai, China) Co., Ltd. The working electrodes for the operando ATR-IR measurements were prepared from a reported method with a little modification^3,53^. Briefly, an Au thin film was chemically deposited onto the reflecting plane of a Si prism, and then drop-coated with the catalyst ink. The Ni_2_P nanoparticles were synthesized by a simple thermal reaction of NaH_2_PO_2_•H_2_O and NiCl_2_•6H_2_O at 250 ^o^C^54^. The electrocatalyst ink was dropped onto Au film to serve as a working electrode (WE) for ATR experiments. The catalyst ink was prepared by mixing 5 mg Ni_2_P powder with 20 uL Nafion solution in 1 mL deionized water and ethanol (volume ratio 1:1). A platinum wire and an Ag/AgCl electrode were used as the counter and reference electrode in all tests, respectively. The 0.1 M KOH with 0.5 M methanol was used as the electrolyte. Before testing, Ni_2_P was activated by applying CV in 0.1 M KOH medium at 100 mV s^−1^ from 0.924 to 1.624 V vs. RHE for five cycles. The chronopotentiometry method was used in this experiment at different potentials (1.35 to 1.65 V vs. RHE without iR-correction). As for IRAS, the ink was loaded on a 5 mm smooth glassy carbon (GC) electrode. The prepared WE was pressed onto the CaF_2_ IR window to collect the spectra.

### Electrochemical measurements

Electrochemical measurements were performed in an H-type cell (H cell) using a CHI760E electrochemical workstation. A Hg/HgO electrode and a graphite rod were used as the reference electrode and counter electrode, respectively. The as-prepared electrodes were used as the working electrode (1 cm × 1 cm). Fumasep FAB-PK-130 was used as an anion exchange membrane (AEM). All the potentials vs. Hg/HgO were converted to the values versus reversible hydrogen electrode (RHE) according to the equation (E vs. RHE = E vs. Hg/HgO + 0.924 V). Linear sweep voltammetry (LSV) polarization curves were recorded at a scan rate of 5 mV s^−1^. All polarization profiles were corrected with 90% iR compensation. Electrochemical impedance spectroscopy (EIS) was conducted on charged catalysts at 0.5 V vs. Hg/HgO over a frequency range from 0.1 to 10 kHz. In situ EIS was carried out by CHI760E in the three-electrode system under different potentials over the frequency range from 1 MHz to 0.1 Hz in 1 M KOH with/without 0.5 M methanol. All experiments were carried out at 25 ^o^C.

### Formate analysis

The methanol oxidative reaction was carried out at 25 ^o^C with stirring by chronoamperometry (I-t) at 1.4, 1.5, 1.6, and 1.7 V vs. RHE for 1 h, respectively. The generated formate at the anode was detected by ion chromatography (IC) (Thermo Scientific Dionex ICS-6000 HPIC).

The yield rate of formate was calculated according to the following formula:

$${{{{{\rm{Formate}}}}}}\,{{{{{\rm{generation}}}}}}=[({{{{{{\rm{C}}}}}}}_{{{{{{\rm{formate}}}}}}}\times {{{{{\rm{V}}}}}})/({{{{{\rm{t}}}}}}\times {{{{{{\rm{M}}}}}}}_{{{{{{\rm{formate}}}}}}})]{{{{{\rm{mol}}}}}}\,{{{{{{\rm{s}}}}}}}^{-1}$$

C_formate_ is the measured formate concentration (g L^−1^) in the solution from the anode compartment of the cell, namely, the IC data. V is the volume of electrolyte (0.03 L), M _formate_ (g mol^−1^) is the molecular weight of formate (HCOO^−^) equal to 45.02 g mol^−1^, and t is the electrolysis time (1 h).

### DFT calculations

The density functional theory calculations were performed by the Vienna ab initio simulation package (VASP) program with the projector augmented wave (PAW) method and the kinetic energy cutoff was set to be 500 eV. Based on the layer feature, NiOOH crystal structures with the termination surface of (000$\bar{1}$) were chosen to build up the periodical models including the substitution of different oxyanions. The thickness of a vacuum to 15 Å in the z-axis was set for the models in order to avoid vertical interaction between nearby slabs. In geometry optimization, the convergences of force and energy were set to 0.02 eV Å^−1^ and 1×10^−5^ eV, respectively. Ni 3*d*-band center and O 2*p*-band center were calculated by integrating the weighted mean energy of the projected density of states (pDOS) of Ni 3*d* and O 2*p* states relative to the Fermi level (E_F_). The five-coordinated Ni atom was selected as the active sites due to sufficient exposure of NiOOH-TO_x_.^55^

The adsorption energies for OH and CH_3_OH on the substrate were described as:

$$\varDelta E({{{{{\rm{OH}}}}}})={{{{{\rm{E}}}}}}({{{{{\rm{sub}}}}}}/{{{{{\rm{OH}}}}}})\mbox{-}[{{{{{\rm{E}}}}}}({{{{{\rm{sub}}}}}})+{{{{{\rm{E}}}}}}({{{{{{\rm{H}}}}}}}_{2}{{{{{\rm{O}}}}}})\mbox{-}1/2\,{{{{{\rm{E}}}}}}({{{{{{\rm{H}}}}}}}_{2})]$$

where E(sub/OH) is the total energies of the OH group on the substrate; E(sub), E(H_2_O), and E(H_2_) denote the total energies of the substrate, H_2_O and H_2_, respectively.

$$\varDelta {{{{{\rm{E}}}}}}({{{{{{\rm{CH}}}}}}}_{3}{{{{{\rm{OH}}}}}})={{{{{\rm{E}}}}}}({{{{{\rm{sub}}}}}}/{{{{{{\rm{CH}}}}}}}_{3}{{{{{\rm{OH}}}}}})\mbox{-}[{{{{{\rm{E}}}}}}({{{{{\rm{sub}}}}}})+{{{{{\rm{E}}}}}}({{{{{{\rm{CH}}}}}}}_{3}{{{{{\rm{OH}}}}}})]$$

where E(sub/CH_3_OH) is the total energies of the CH_3_OH molecule on the substrate; E(sub) and E(CH_3_OH) denote the total energies of the substrate, and CH_3_OH, respectively.

## Supplementary information

Supplementary Information

## Data Availability

Additional data related to this study are available from the corresponding authors on reasonable request. Source data are provided with this paper.

## Source data

Source Data
