# Supplementary material for: Coordination environment tuning of nickel sites by oxyanions to optimize methanol electro-oxidation activity
Source: Nat Commun. 2022 May 25;13:2916. doi: 10.1038/s41467-022-30670-4 (PMC9133001; doi:10.1038/s41467-022-30670-4)
Supplement: Supplementary file 1 — Supplementary Information [file 41467_2022_30670_MOESM1_ESM.pdf]

Supplementary information

Coordination Environment Tuning of Nickel Sites by Oxyanions to Optimize Methanol Electro-oxidation Activity

Shanlin Li[1,2,3,#], Ruguang Ma[1,4,#], Jingcong Hu[3], Zichuang Li[1,2], Lijia Liu[5], Xunlu Wang[1,2], Yue Lu[3], George E. Sterbinsky [6], Shuhu Liu[7], Lei Zheng[7], Jie Liu[1], Danmin Liu[3], Jiacheng Wang[1,2,8*]

1. The State Key Laboratory of High Performance Ceramics and Superfine Microstructure, Shanghai Institute of Ceramics, Chinese Academy of Sciences, Shanghai 200050, China

Corresponding author: E-mail: jiacheng.wang@mail.sic.ac.cn

2. Center of Materials Science and Optoelectronics Engineering, University of Chinese Academy of Sciences, Beijing 100049, China

3. Beijing Key Laboratory of Microstructure and Properties of Solids, Faculty of Materials and Manufacturing, Beijing University of Technology, Beijing 100124, China

4. School of Materials Science and Engineering, Suzhou University of Science and Technology, 99 Xuefu Road, Suzhou 215011, China

5. Department of Chemistry, Western University, 1151 Richmond Street, London, ON N6A5B7, Canada

6. Advanced Photon Source, Argonne National Laboratory, Argonne, IL 60439, USA

7. Institute of High Energy Physics, Chinese Academy of Sciences, Beijing 100049, China

8. Hebei Provincial Key Laboratory Nonmetallic Materials, College of Materials Science and Engineering, North China University of Science and Technology, Tangshan 063210, China

#. These authors contributed equally.

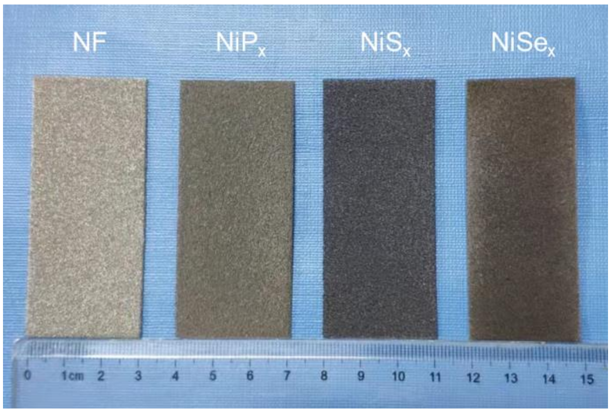

Supplementary Fig. 1 Photos of samples.

Optical photos of NF and NiT_x.

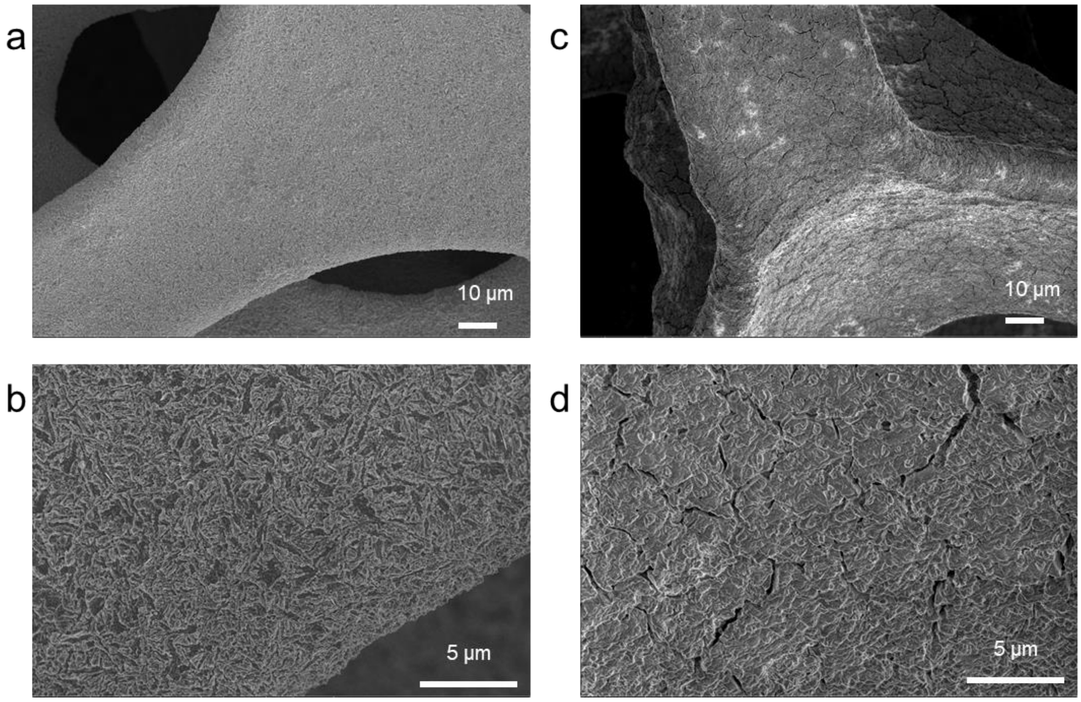

Supplementary Fig. 2 Morphology characterization of NiP_x and NiP_x-R.
SEM images of NiP_x (a-b) and NiP_x-R (c-d).

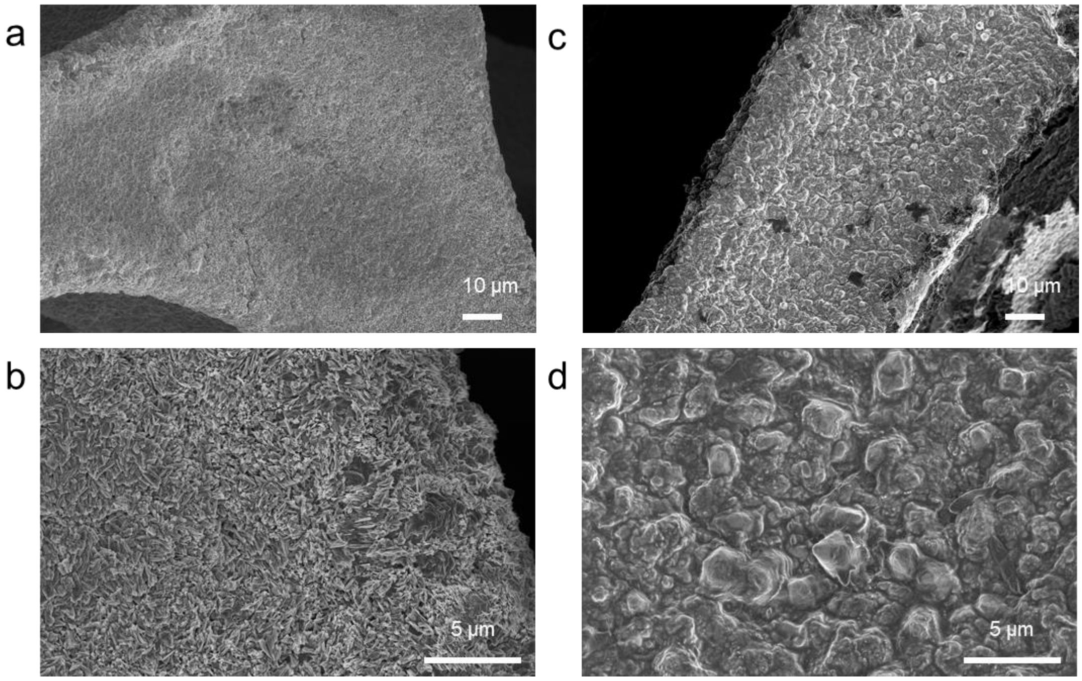

Supplementary Fig. 3 Morphology characterization of NiS_x and NiS_x-R.
SEM images of NiS_x (a-b) and NiS_x-R (c-d).

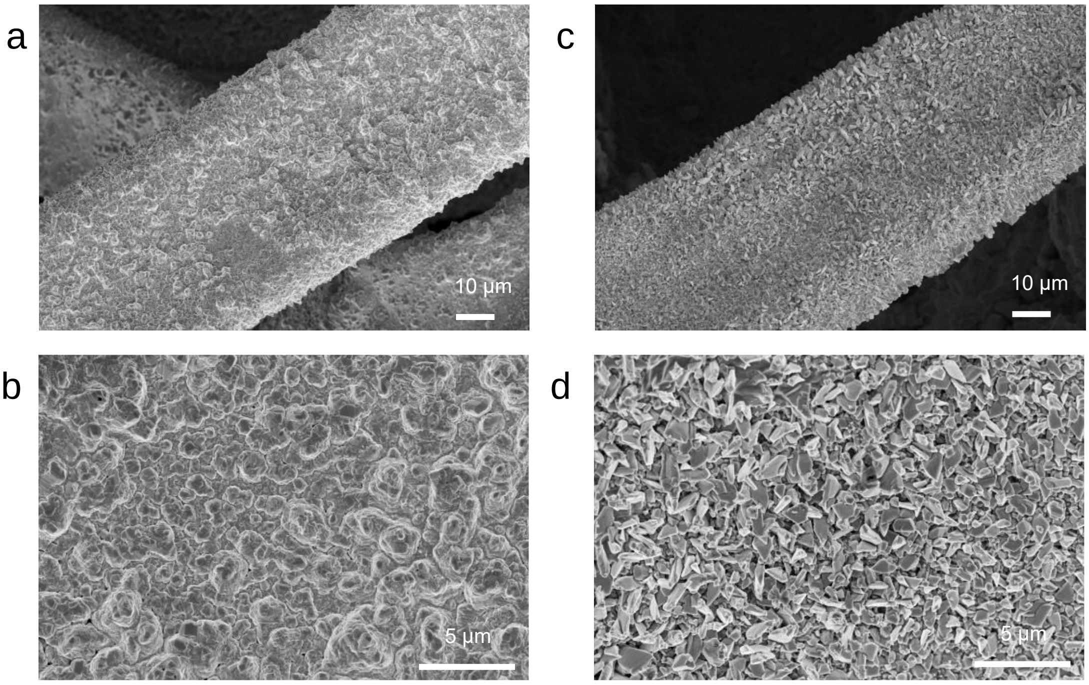

Supplementary Fig. 4 Morphology characterization of $NiSe_x$ and $NiSe_x$-R. SEM images of $NiSe_x$ (a-b) and $NiSe_x$-R (c-d).

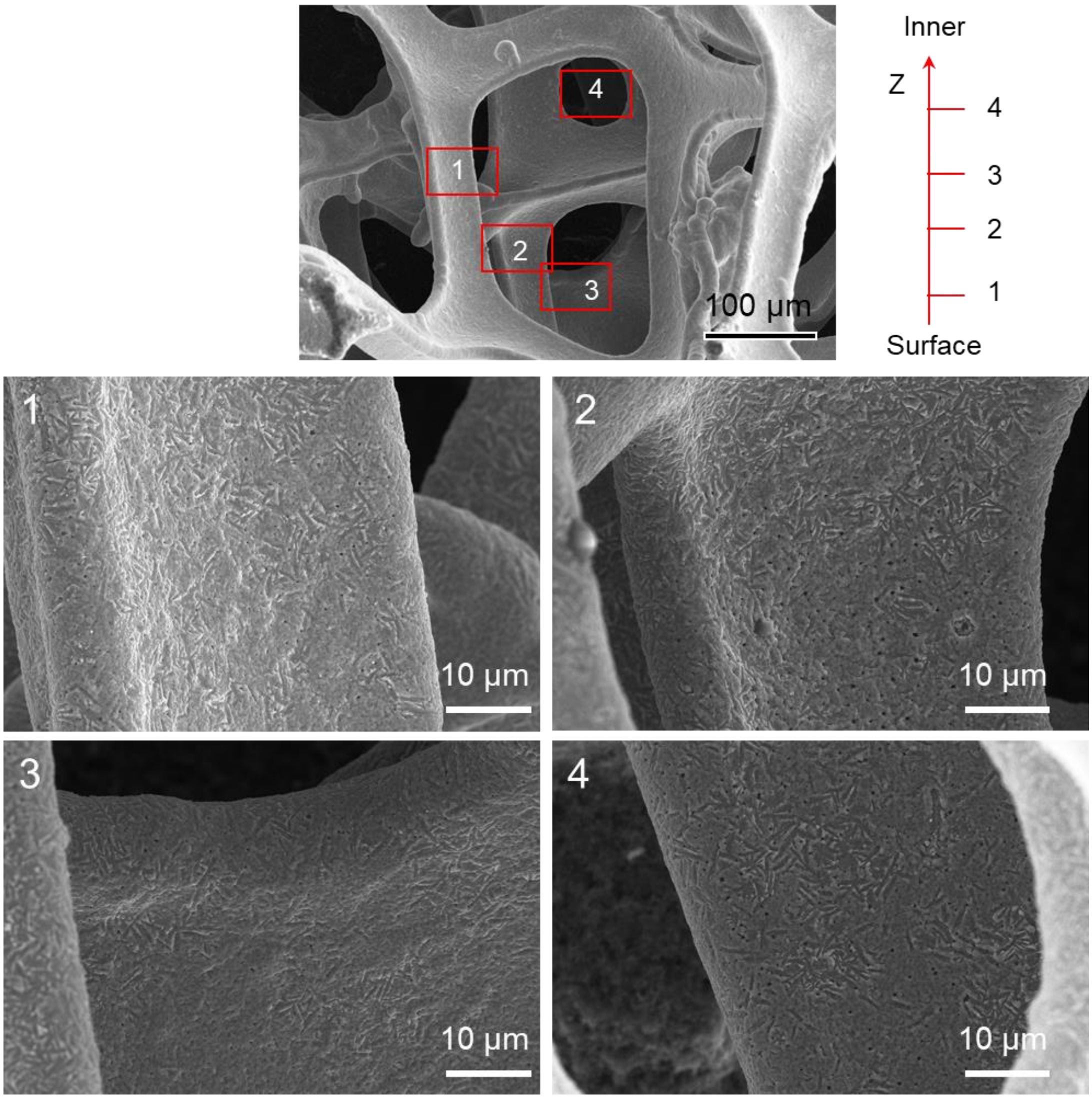

Supplementary Fig. 5 Morphology characterization of NiP_x.
SEM images of NiP_x at different focal depth. There is no significant topographic change with the focal plane, which proves that the inner part is also fully covered.

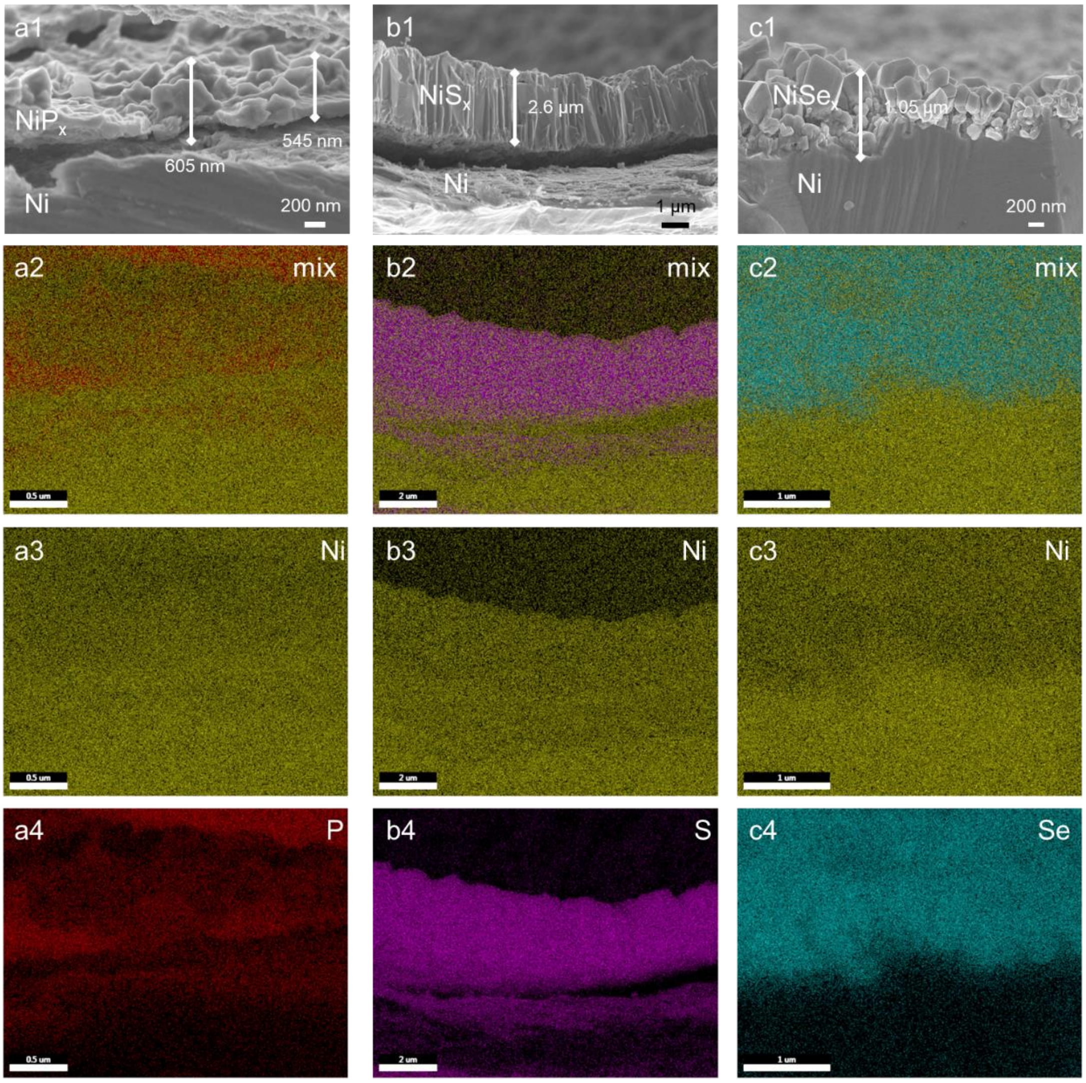

Supplementary Fig. 6 Morphology and compositional characterization of NiT_x. Cross-section SEM images (a1-c1) and elemental mapping (a2-a4, b2-b4, and c2-c4) of NiP_x (a), NiS_x (b) and $NiSe_x$ (c).

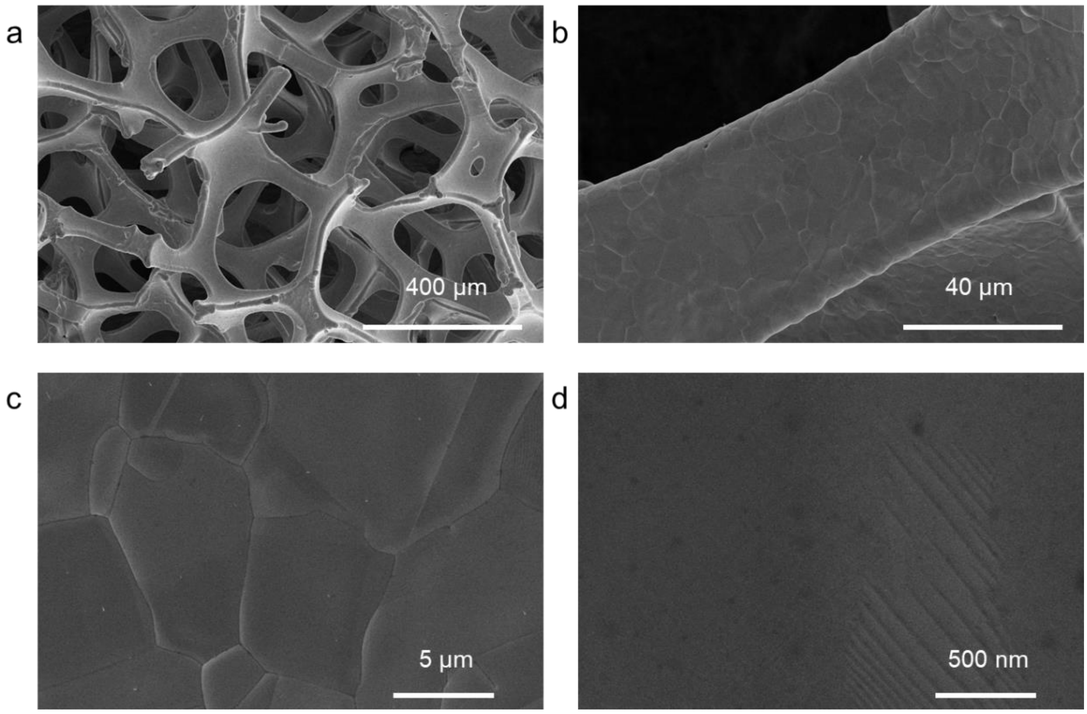

Supplementary Fig. 7 Morphology characterization of Ni foam (NF).
SEM images of fresh NF at different magnifications: 100X(a), 1000X (b), 5000X(c) and 50000X(d).

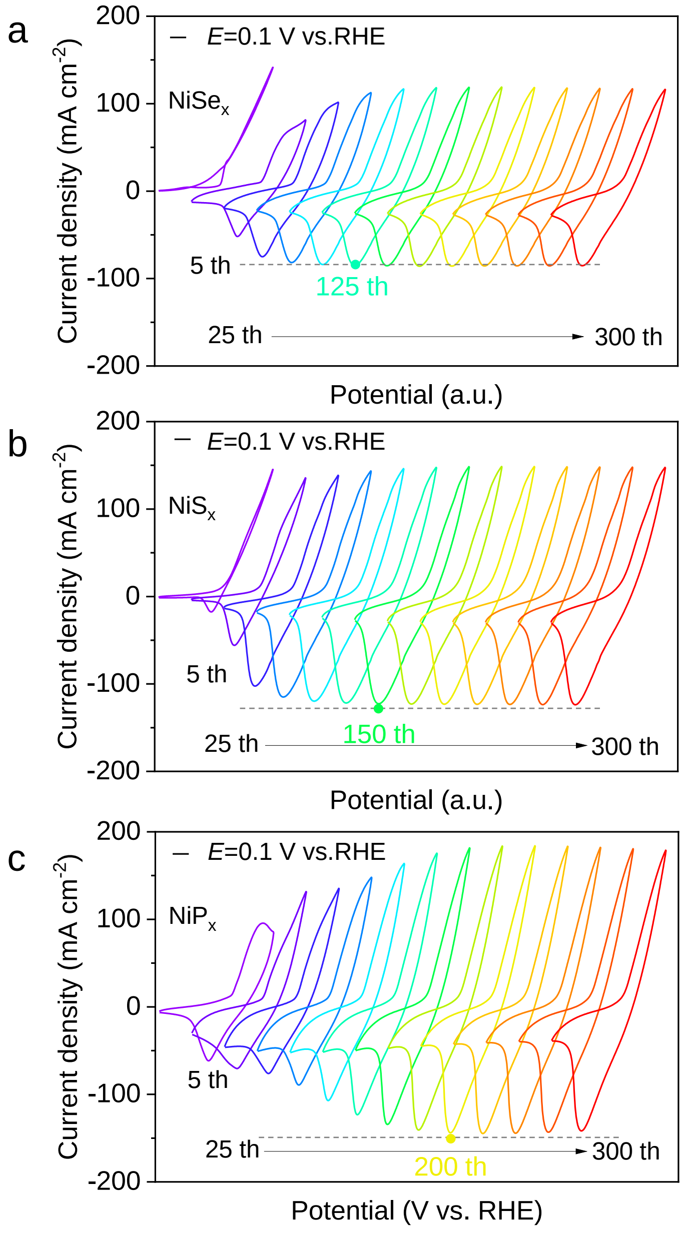

Supplementary Fig. 8 Electrochemical surface reconstruction.
Evolution of CV curves for (a) NiP_x, (b) NiS_x, (c) $NiSe_x$ from the 5th to the 300th cycle in 1 M KOH at 100 mV s^{-1}.

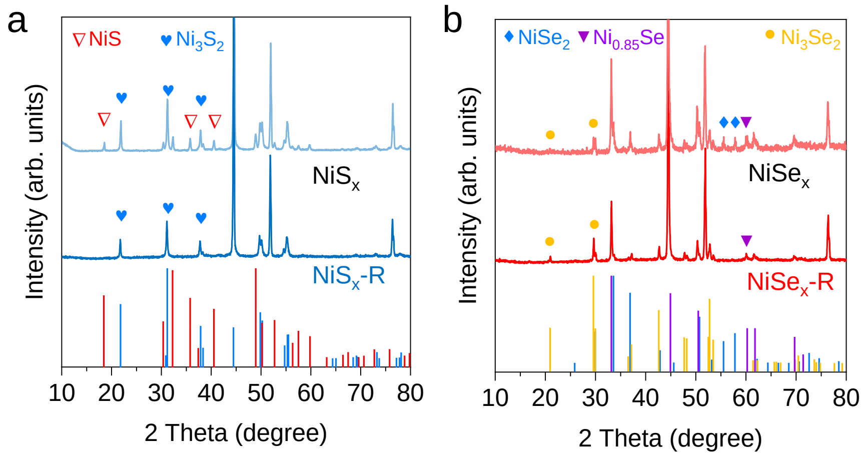

Supplementary Fig. 9 XRD characterizations.

XRD patterns for (a) NiS_x and NiS_x-R, and (b) $NiSe_x$ and $NiSe_x$-R.

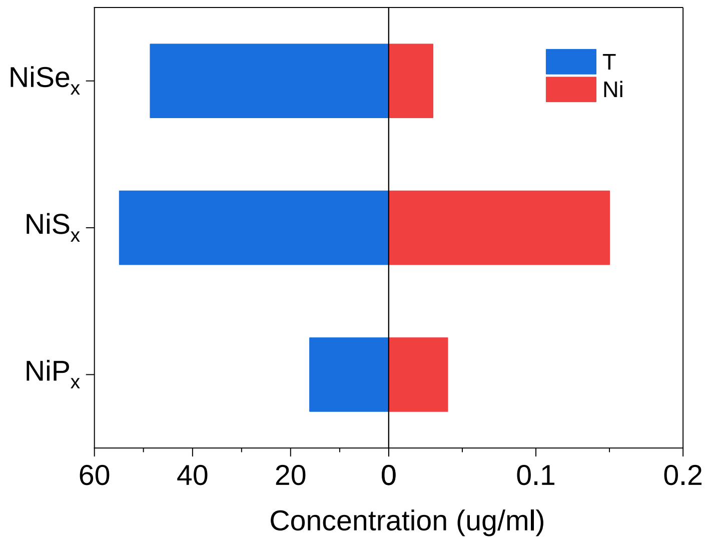

Supplementary Fig. 10 Determination of dissolved elements in electrolyte. ICP-OES analysis of the electrolyte for NiT_x (T = P, S, or Se) after surface reconstruction.

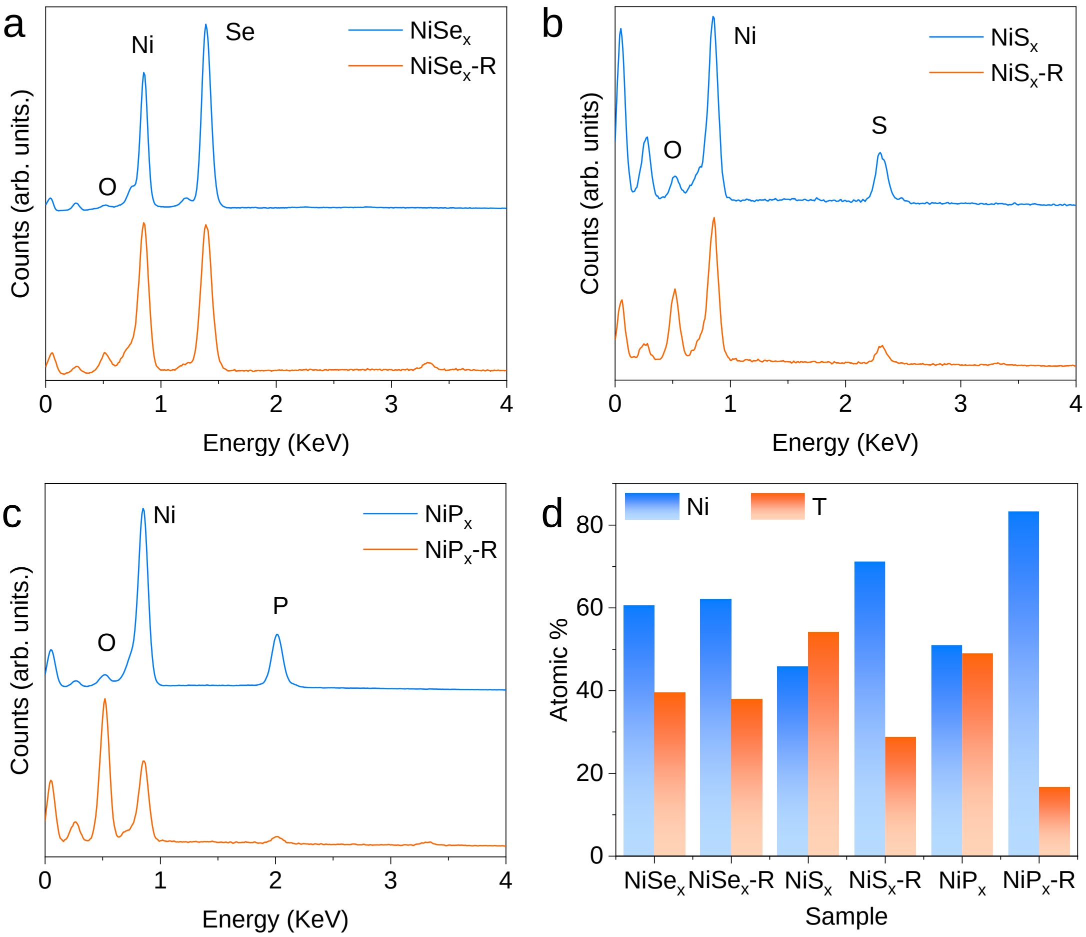

Supplementary Fig. 11 Scanning electron microscopy–energy-dispersive X-ray spectroscopy (SEM-EDS) analysis.

SEM-EDS spectra of (a) $NiSe_x$ and $NiSe_x$-R, (b) NiS_x and NiS_x-R, and (c) NiP_x and NiP_x-R. (d) The atomic percentage of Ni and T in $NiSe_x$, $NiSe_x$-R, NiS_x, NiS_x-R, NiP_x and NiP_x-R.

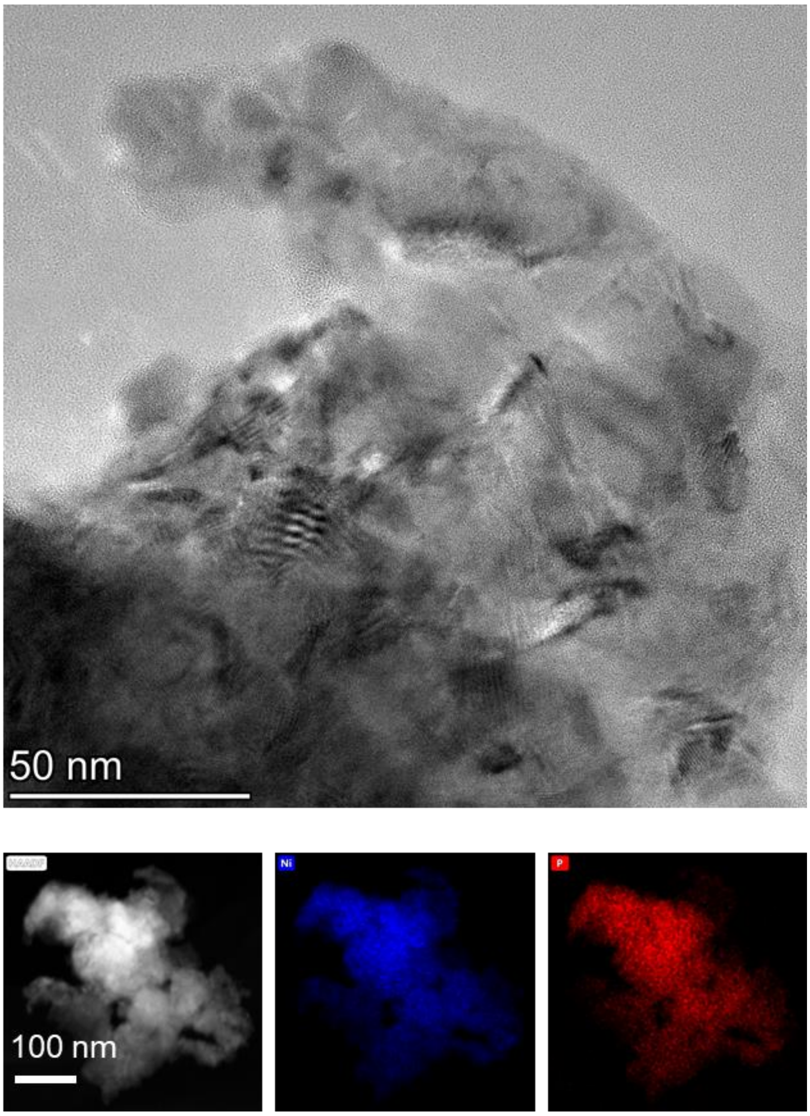

Supplementary Fig. 12 Morphology and compositional characterization of NiP_x by TEM.
TEM and EDS elemental mapping images of NiP_x.

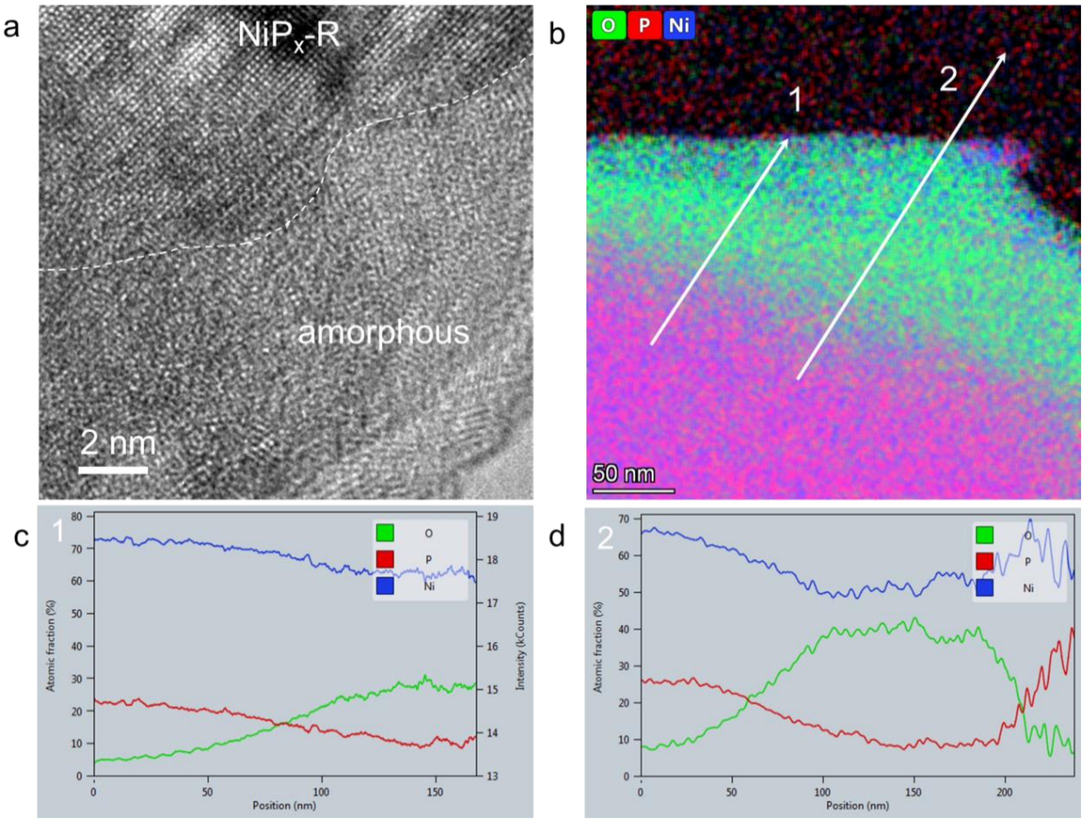

Supplementary Fig. 13 Structural and compositional characterizations of NiP_x-R. (a) HRTEM image, (b) EDS elemental mapping, and (c-d) EDS line scanning results of NiP_x-R.

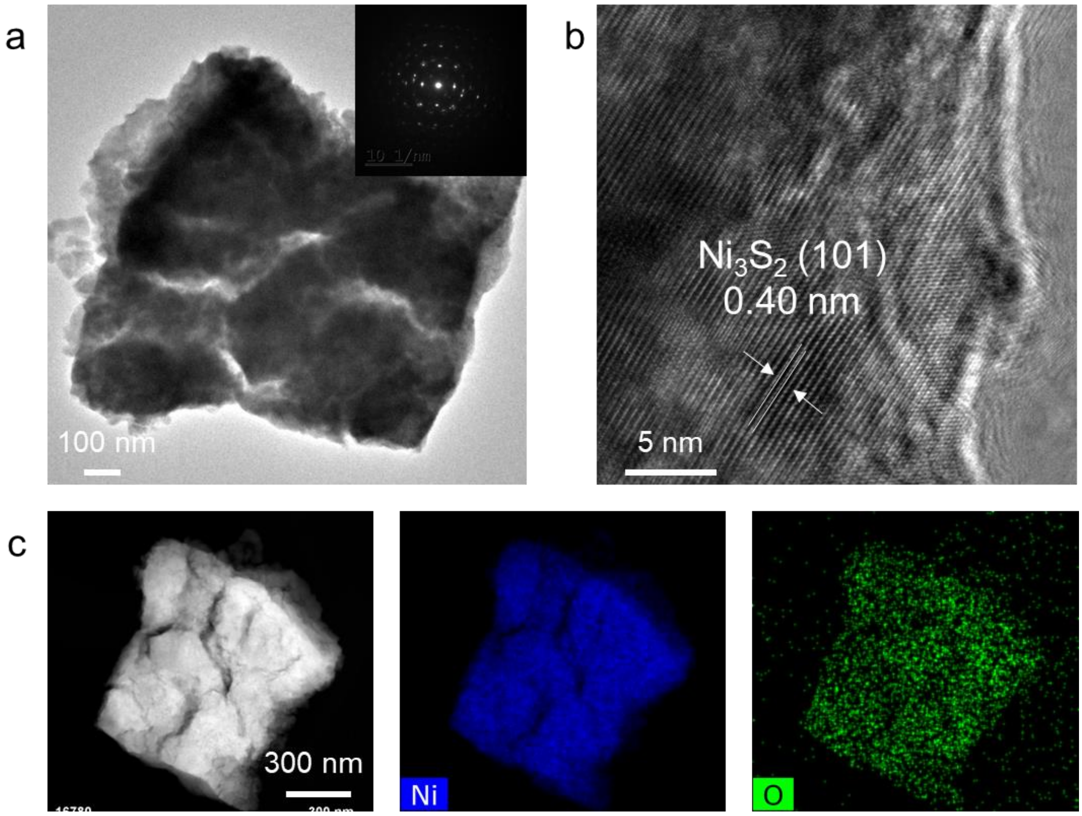

Supplementary Fig. 14 Morphology and compositional characterization of NiS_x
(a) TEM image, (b) HRTEM image and (c) EDS elemental mapping of NiS_x.

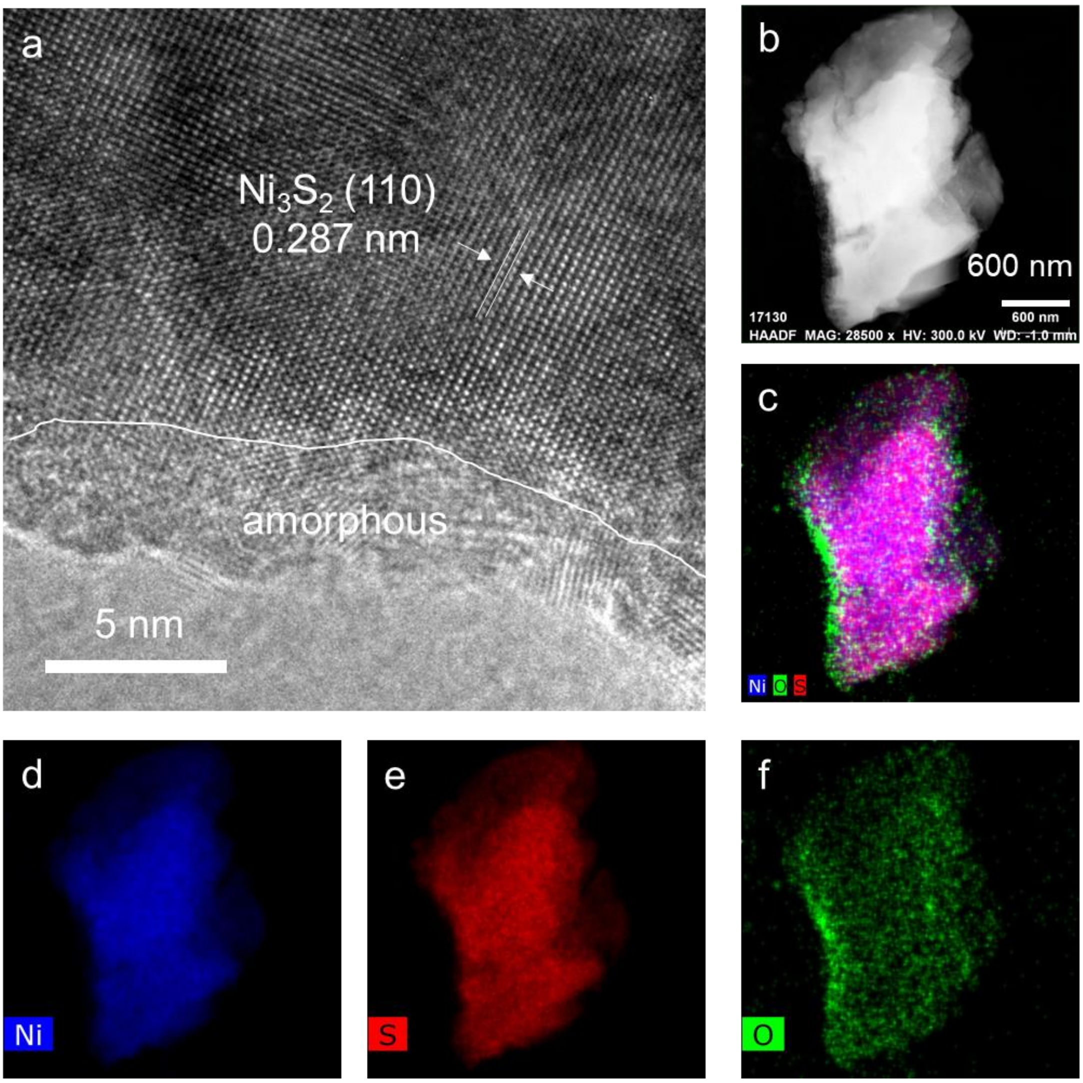

Supplementary Fig. 15 Structural and compositional characterizations of NiS_x-R. (a) HRTEM image, (b) HADDF-STEM image and (c-f) HAADF and EDS elemental mapping of NiS_x-R.

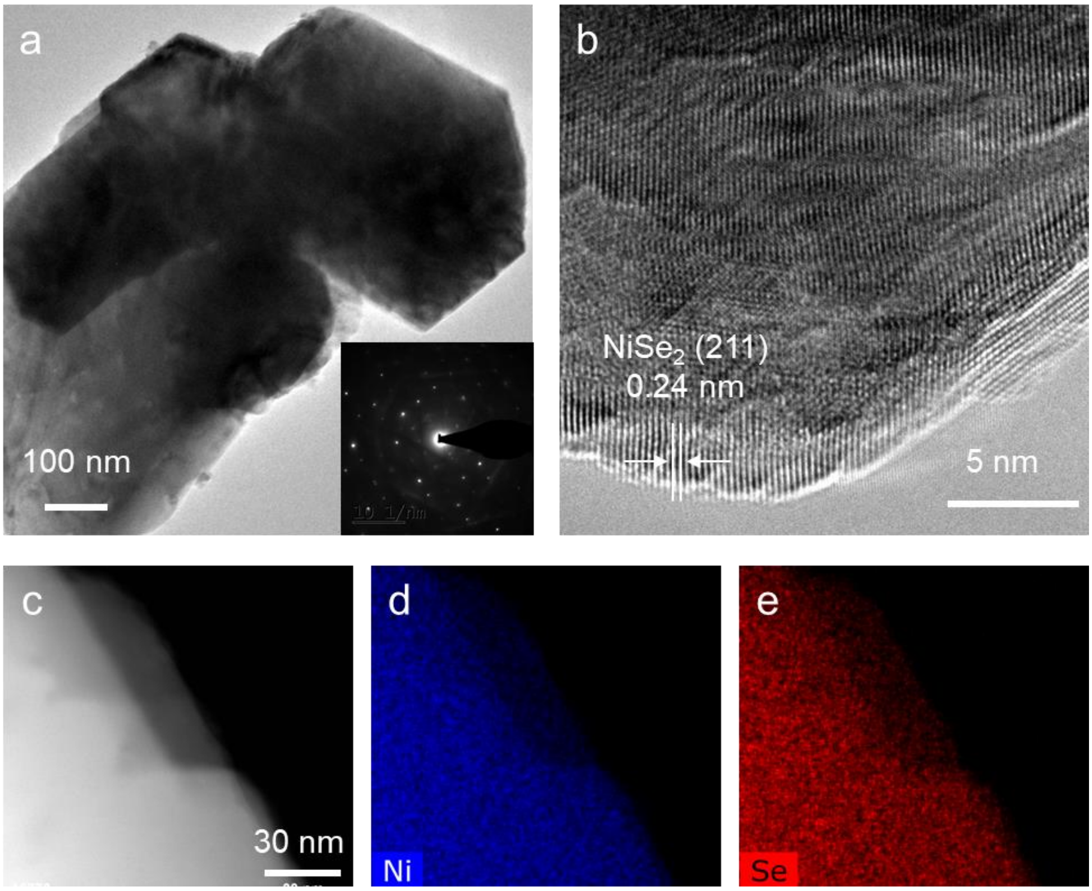

Supplementary Fig. 16 Morphology and compositional characterization of $NiSe_x$.
(a) TEM image, (b) HRTEM image and (c-e) HAADF and EDS elemental mapping of $NiSe_x$.

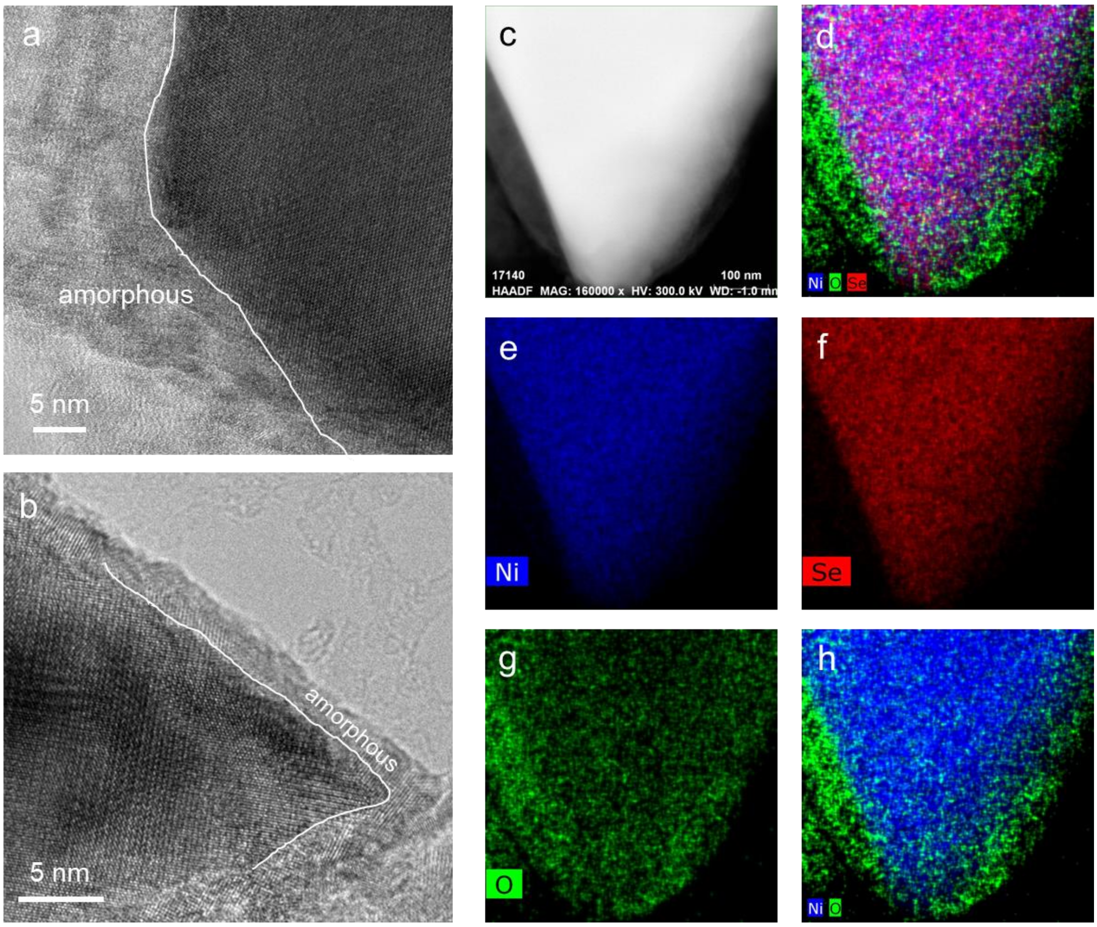

Supplementary Fig. 17 Structural and compositional characterizations of $NiSe_x$-R. (a) TEM image, (b) HRTEM image and (c-h) HAADF and EDS elemental mapping images of $NiSe_x$-R.

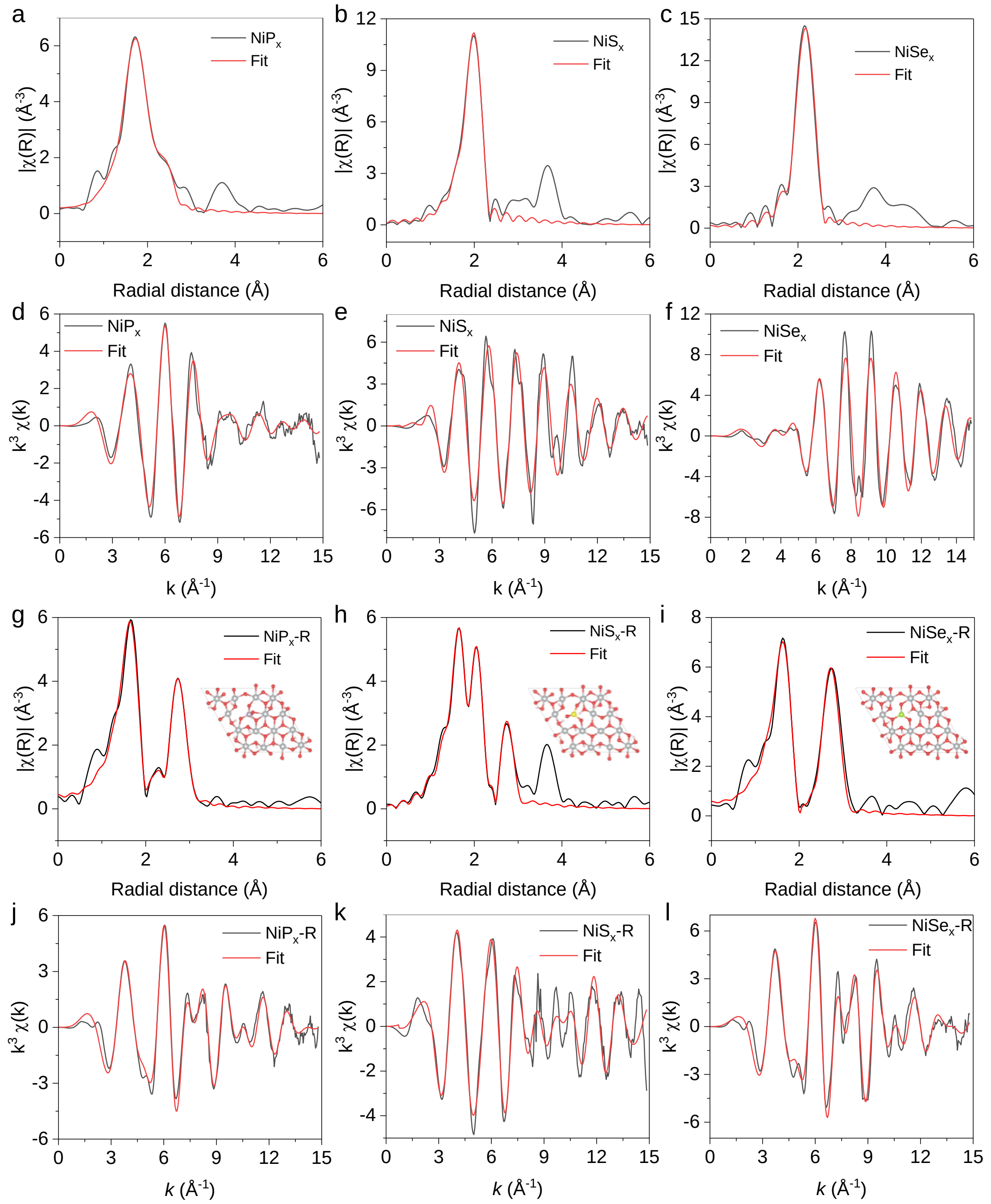

Supplementary Fig. 18 EXAFS fitting results.

The EXAFS R-space fitting curves (red line) and the experimental one (black line) of NiP_x(a), NiS_x(b) and $NiSe_x$(c). The EXAFS k-space fitting curves (red line) and the experimental one (black line) of NiP_x(d), NiS_x(e) and $NiSe_x$(f). The EXAFS R-space fitting curves (red line) and the experimental one (black line) of NiP_x-R(g), NiS_x-R (h) and $NiSe_x$-R (i). The EXAFS k-space fitting curves (red line) and the experimental one (black line) of NiP_x-R (j), NiS_x-R (k) and $NiSe_x$-R (l).

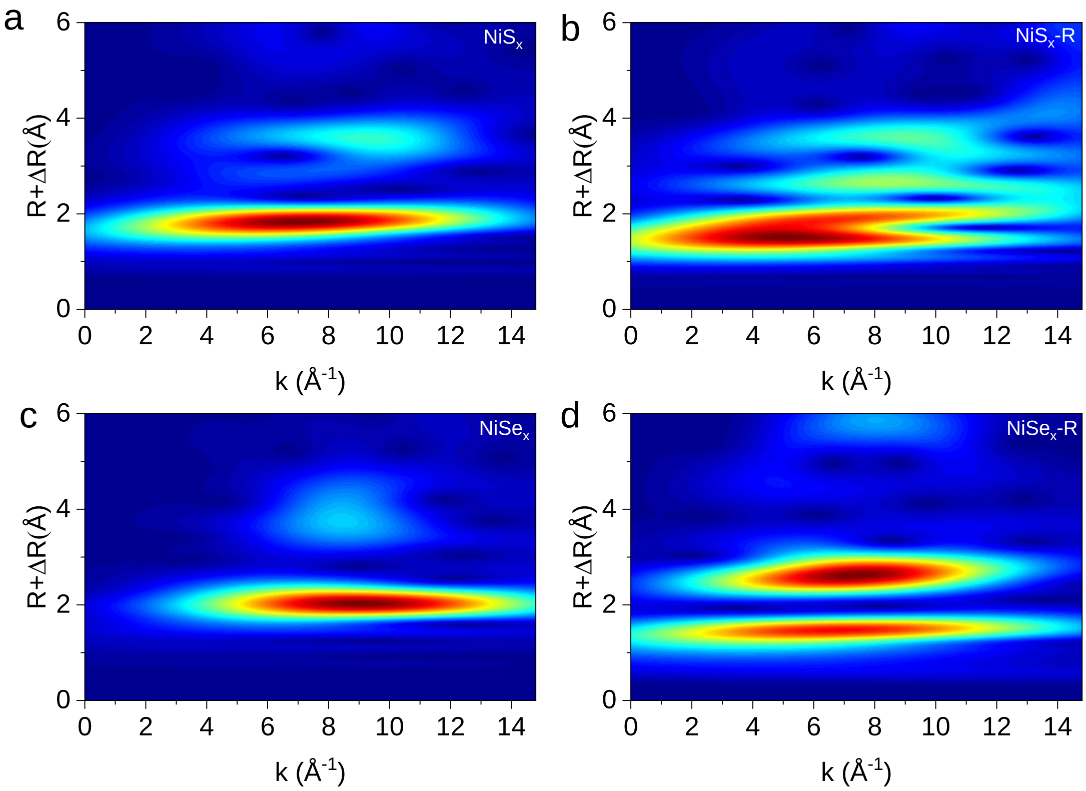

Supplementary Fig. 19 WT-EXAFS results.

WT-EXAFS at Ni K-edge for (a) NiS_x, (b) NiS_x-R, (c) $NiSe_x$, and (d) $NiSe_x$-R.

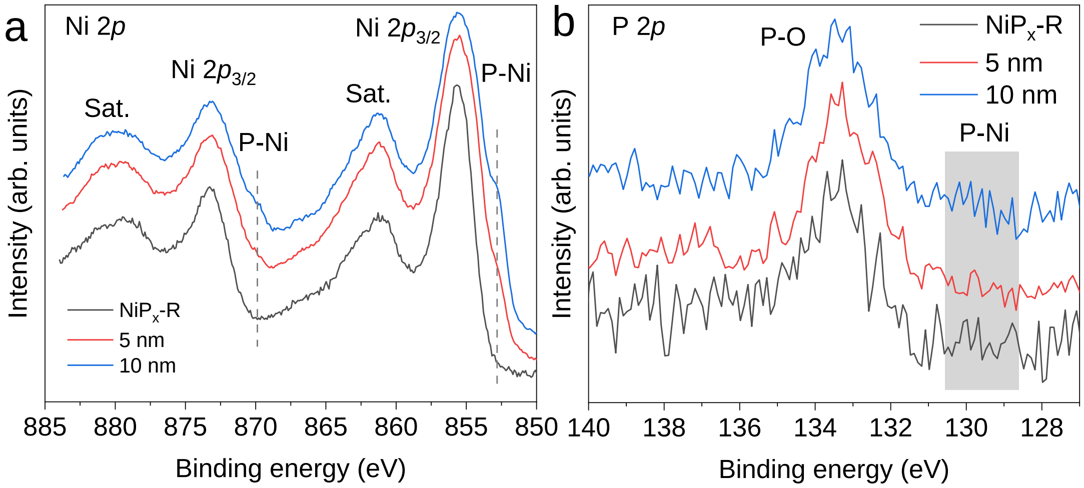

Supplementary Fig. 20 Surface chemical state characterization before and after etching.

High-resolution depth-profiling XPS spectra of Ni 2*p* (a) and P 2*p* (b) for NiP_x-R.

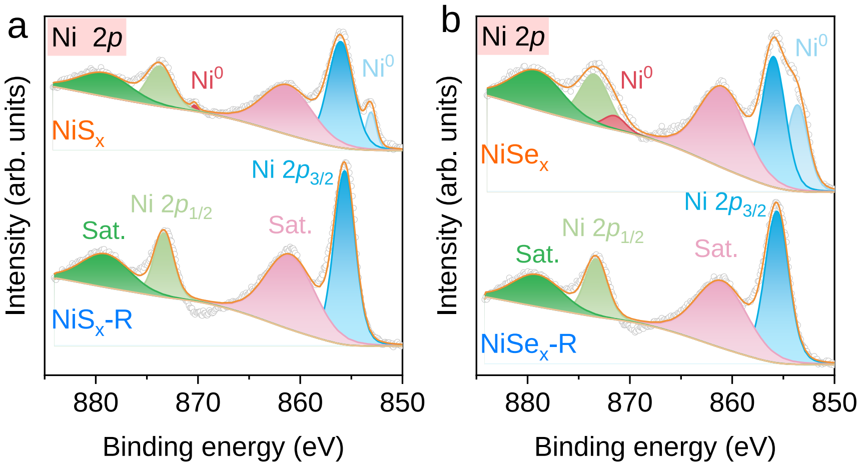

Supplementary Fig. 21 XPS characterization.

High-resolution Ni 2*p* XPS spectra of NiS_x and NiS_x-R (a), $NiSe_x$ and $NiSe_x$-R (b).

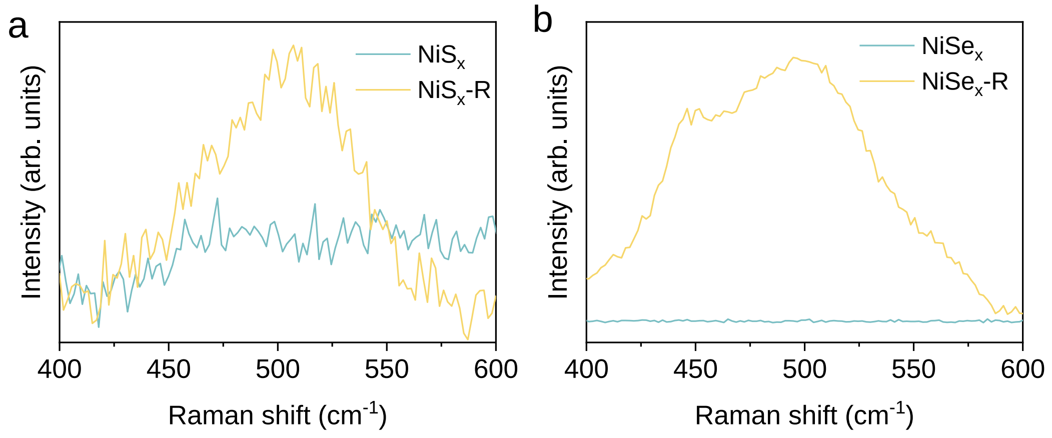

Supplementary Fig. 22 Raman characterization.

Raman spectra of NiS_x and NiS_x-R (a), $NiSe_x$ and $NiSe_x$-R (b).

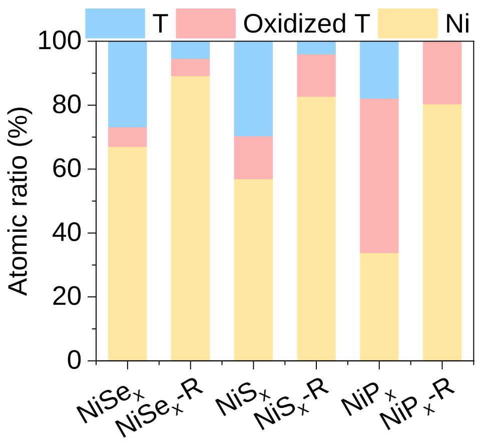

Supplementary Fig. 23 XPS analysis.

Atomic ratio variation of NiT_x and NiT_x-R measured by XPS.

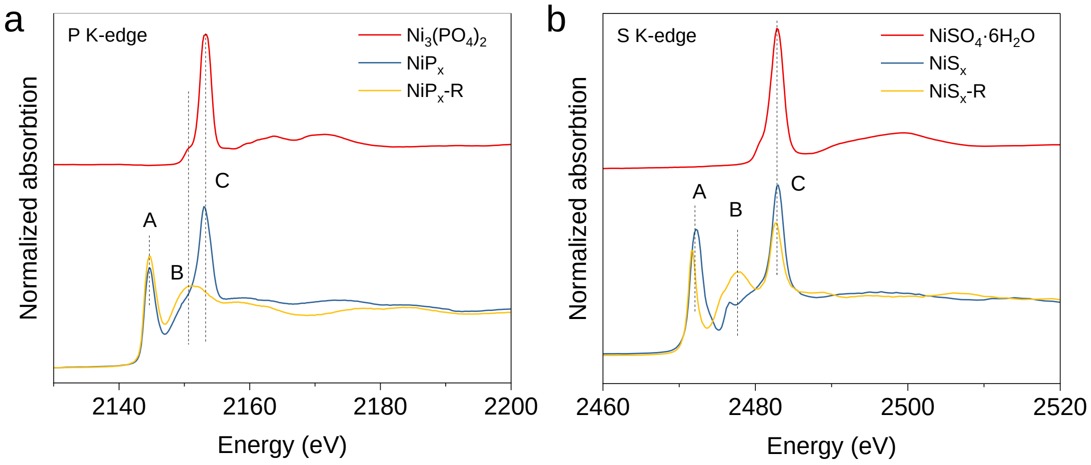

Supplementary Fig. 24 XANES characterization of P and S.

(a) P K-edge XANES spectra of NiP_x, NiP_x-R and $Ni_3(PO_4)_2$, (b) S K-edge XANES spectra of NiS_x, NiS_x-R and $NiSO_4$•$6H_2O$.

Supplementary Fig. 24a displays the P K-edge XANES spectra for NiP_x, NiP_x-R, and $Ni_3(PO_4)_2$ standard material. The spectra exhibit three major features which are centered at ~ 2144 eV (A: P(1*s*) → P(3*d*) and Ni (3*d*/4*d*)), ~2150 eV (B: pre-edge; P(1*s*) → P(3*d*) and Ni (3*d*)), 2153 eV (C: white line; P (1*s*) → P (3*p*) and O (2*p*)). The first absorption peak A is assigned to the Ni-P resonance of Ni_2P. (Ref: (1) Franke R. X-ray absorption and photoelectron spectroscopy investigation of binary nickel phosphides. *Spectrochimica Acta Part A: Molecular and Biomolecular Spectroscopy* **53**, 933-941 (1997). (2) Blanchard PER, Grosvenor AP, Cavell RG & Mar A. X-ray Photoelectron and Absorption Spectroscopy of Metal-Rich Phosphides M_2P and M_3P (M = Cr−Ni). *Chem. Mater.* **20**, 7081-7088 (2008)). The peak C at 2153 eV matches well with the $Ni_3(PO_4)_2$ standard, and is consistent with the presence of a phosphate species in NiP_x. The absorption peak B of NiP_x-R is consistent with a phosphorus species having an oxidation state intermediate between phosphide and phosphate. (Ref: Saadi FH, et al. Operando Spectroscopic Analysis of CoP Films Electrocatalyzing the Hydrogen-Evolution Reaction. *J. Am. Chem. Soc.* 139, 12927-12930 (2017)). The pre-edge peak B at around 2150 eV is related to the hybridization of P 3*p* electrons with the incomplete Ni 3*d* electronic layer of the conduction band associated with a PO-Ni bond / PO(OH)-Ni bonding (Nickel oxy-hydroxide phase to phosphorus) bond (Ref: Gurbani

N, *et al.* Graphene oxide@nickel phosphate nanocomposites for photocatalytic hydrogen production. *Chemical Engineering Journal Advances* **6**, 100105 (2021)). The above results showed the presence of the inorganic P-O-Ni species after the reconstruction in NiP_x-R.

Supplementary Fig. 24b shows the S K-edge XANES spectra of $NiSO_4$•H_2O and NiS_x before and after the reconstruction. The position of the S K-edge is sensitive to the oxidation state. The white-line peak shifts to high energy by 10-13 eV from semiconducting sulfides (S^{2-}) to sulfates (SO_4^{2-}) (Ref: (1) Struis RPWJ*, et al.* Sulphur poisoning of Ni catalysts in the SNG production from biomass: A TPO/XPS/XAS study. *Appl. Catal. A* **362**, 121-128 (2009); (2) Farrell SP&Fleet ME. Sulfur K-edge XANES study of local electronic structure in ternary monosulfide solid solution [(Fe, Co, Ni) 0.923 S]. *Phys. Chem. Miner.* **28**, 17-27 (2001)). Peak A of NiS_x and NiS_x-R can be assigned to the S-Ni bonding. The peak C at ~2483 eV is ascribed to SO_4^{2-}, which matches with $NiSO_4$•H_2O standard. The new peak B of NiS_x-R means the presence of new sulfur species with an oxidation state between 2 and 6.

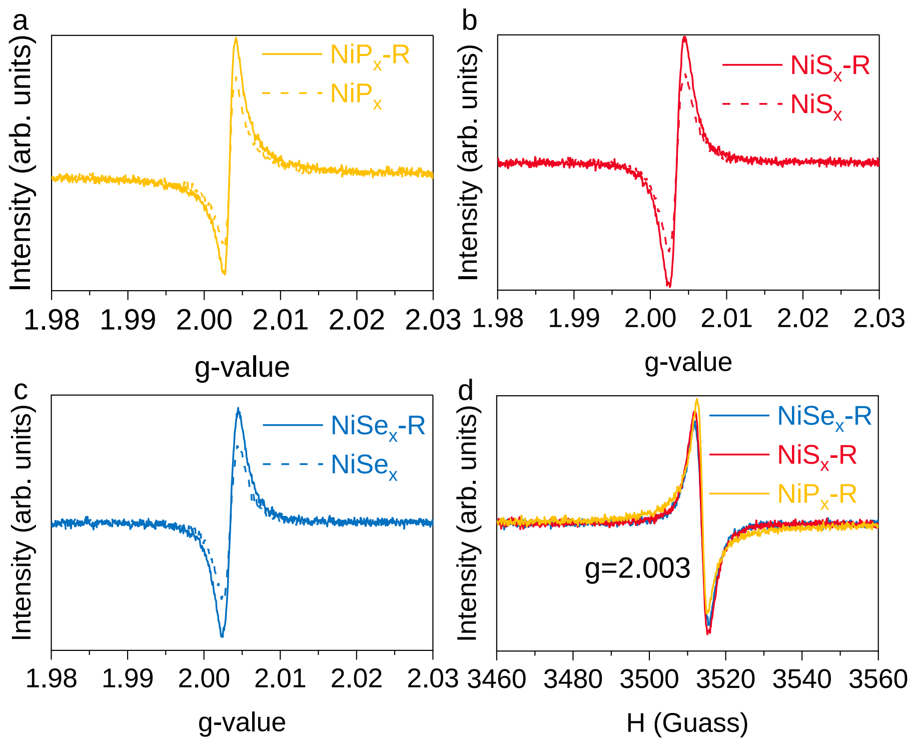

Supplementary Fig. 25 Oxygen vacancy characterization.

EPR spectra of (a) NiP_x and NiP_x-R, (b) NiS_x and NiS_x-R, (c) $NiSe_x$ and $NiSe_x$-R, (d) NiP_x-R, NiS_x-R and $NiSe_x$-R.

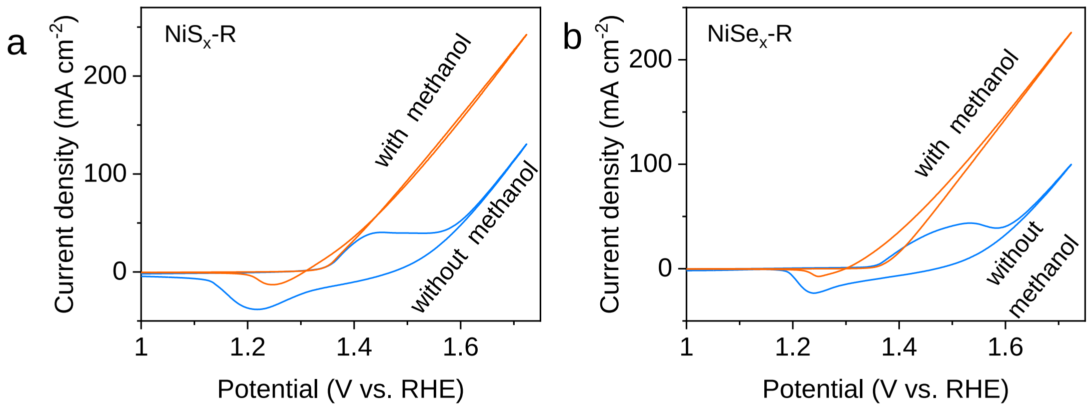

Supplementary Fig. 26 Electrochemical characterization.

Cyclic voltammetry (CV) curves of NiS_x-R (a) and $NiSe_x$-R (b) in 1 M KOH solution at a scan rate of 5 mV s^{-1} with and without 0.5 M methanol.

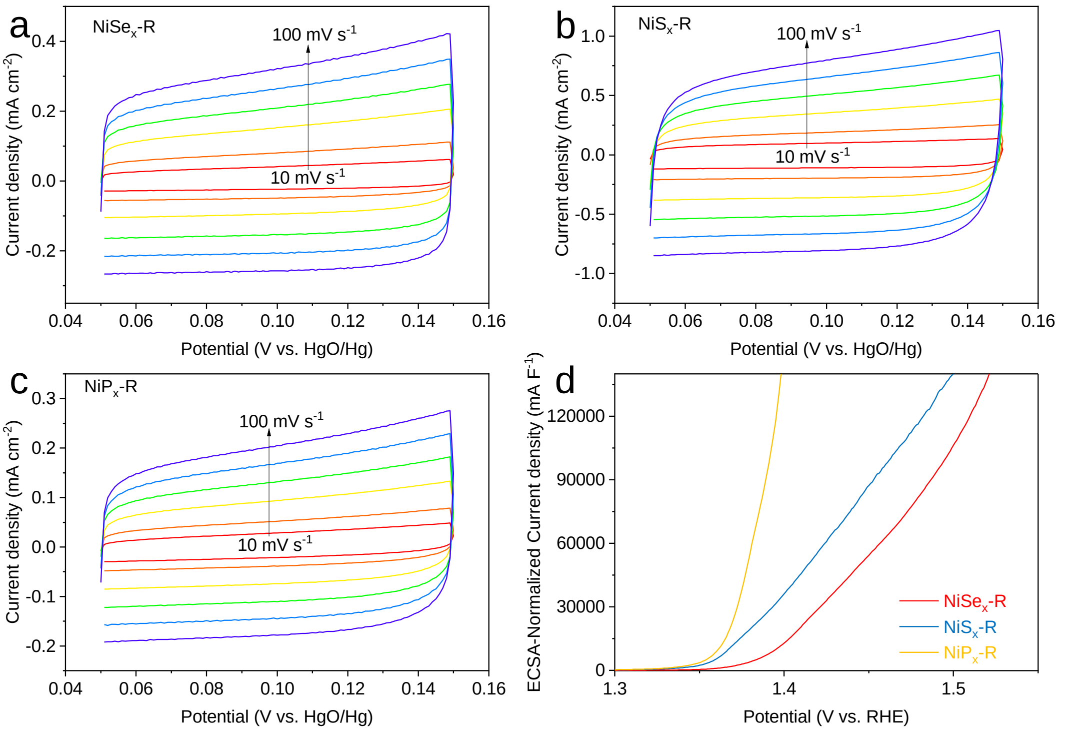

Supplementary Fig. 27 CV curves with different scan rates.

(a-c) Double-layer capacitance (C_{dl}) values extracted from cyclic voltammetry (CV) measurements in non-Faradaic regions. The CV measurements are performed in 1.0 M KOH solution with 0.5 M methanol. (d) Double layer capacitance normalized-LSVs of NiP_x-R, NiS_x-R and $NiSe_x$-R toward MOR.

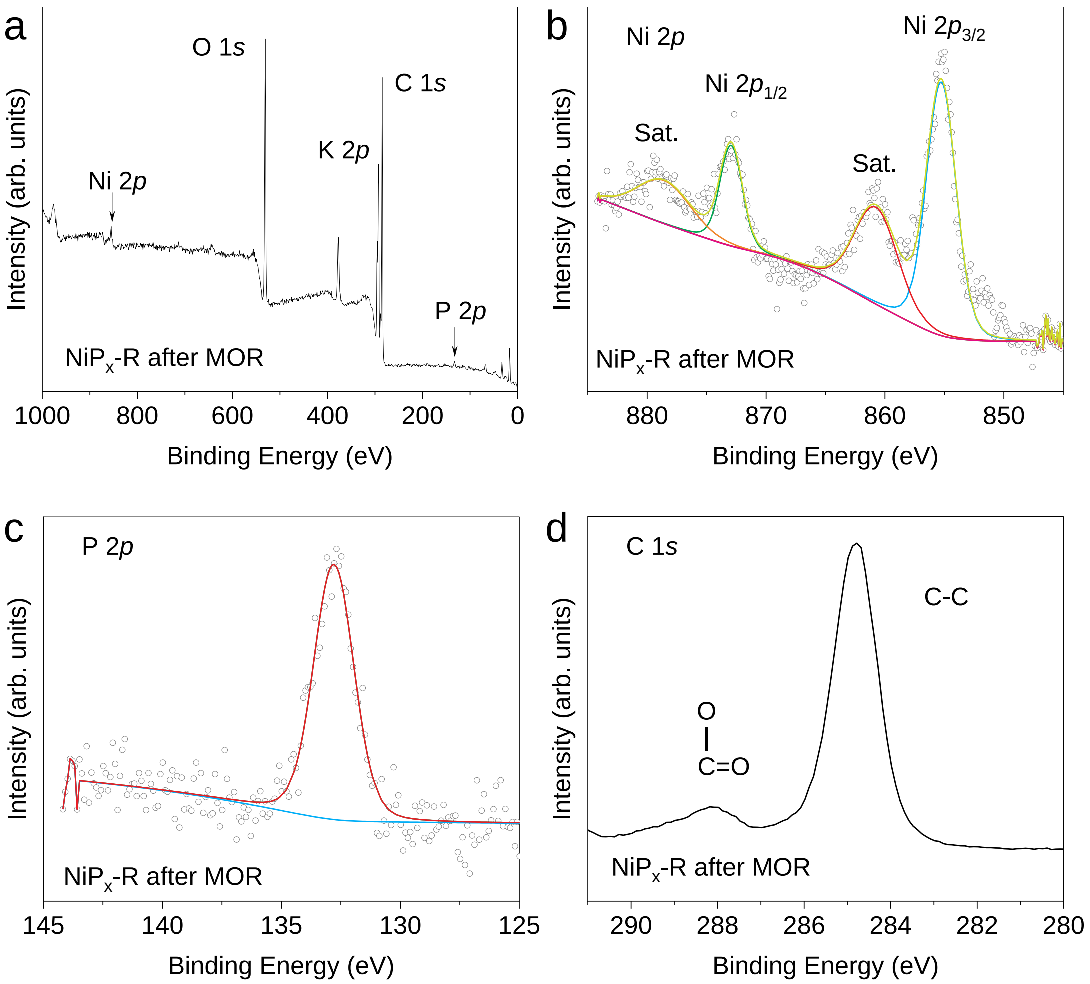

Supplementary Fig. 28 XPS analysis of NiP_x-R after MOR.

XPS survey spectra (a) and high-resolution XPS spectra in the Ni 2*p* (b), P 2*p* (c) and C 1*s* (d) regions for the used NiP_x-R after MOR.

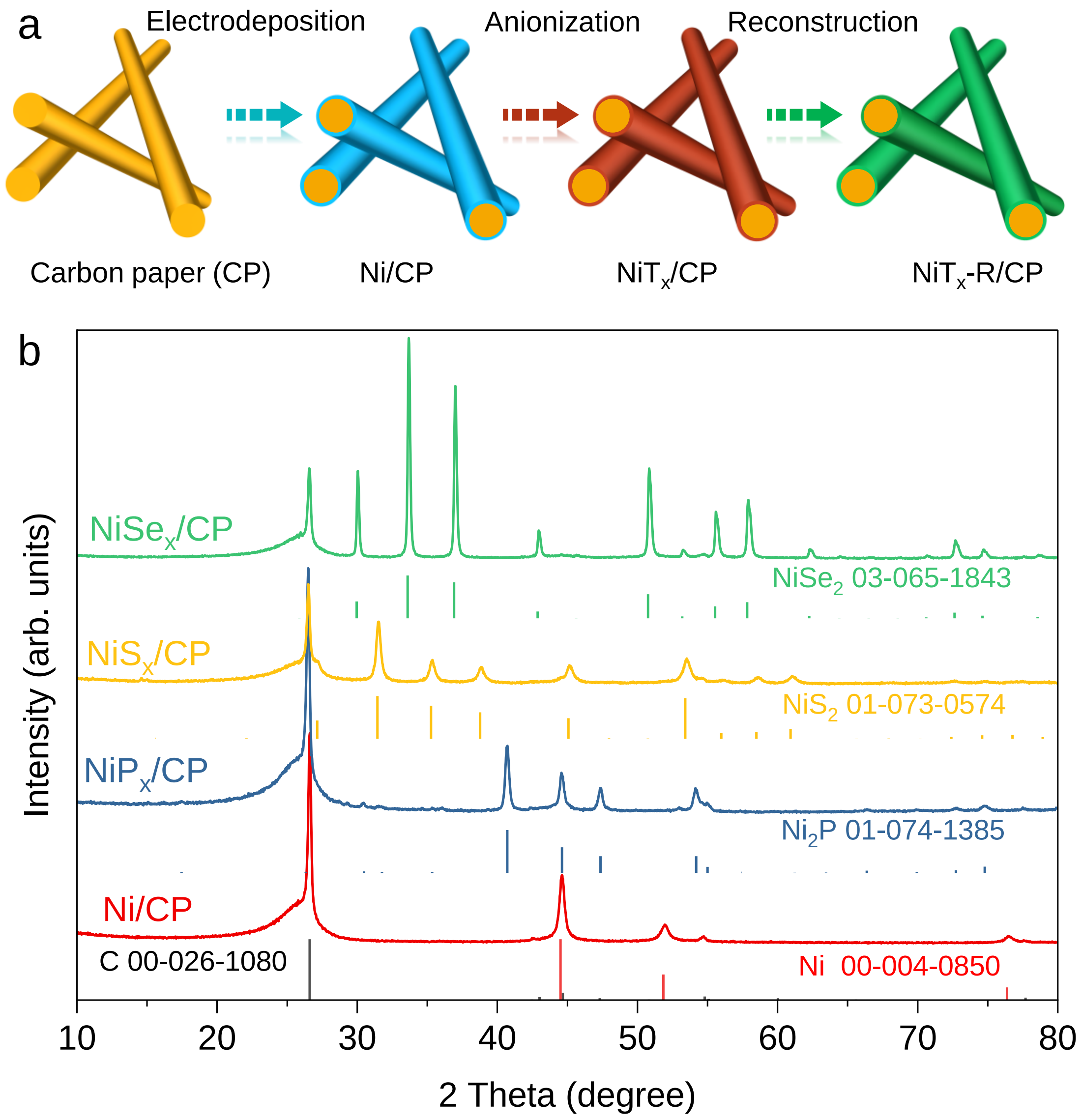

Supplementary Fig. 29 Synthesis and characterization of NiT_x/CP.

(a) Schematic drawing of the synthesis route of NiT_x-R on carbon paper. (b) XRD patterns for Ni/CP, NiP_x/CP, NiS_x/CP and $NiSe_x$/CP.

To exclude the effect of NF substrate and NiT_x thickness on the final MOR performance, we added the experiments (Supplementary Fig. 29a). Firstly, we specifically deposited nickel with the same thickness on carbon paper (CP) by electrodeposition, and further used the anionization method in our article to completely convert nickel into different NiTx. After the same electrochemical reconstruction, we tested MOR performance of carbon paper samples. We found that NiT_x samples with the same thickness still exhibit different MOR performance after reconstruction, and the NiP_x-R is still the best MOR catalyst.

Considering that NiT_x is prepared by anionization using NF as the nickel source, the thickness of NiT_x cannot be controlled because the nickel source is always in excess. We propose to deposit the same thickness of nickel coating on carbon paper (CP) by electrodeposition to prepare NiT_x with the same thickness. First, Ni was then coated on the CP (1×2 cm^2) through electrodeposition. With the CP acting as the working electrode and Pt as the counter electrode, a constant current of -5 mA cm^{-2} was obtained for 900 seconds. The electrodeposition solution contained 0.15 M $NiSO_4$ and 0.6 M H_3BO_4. After electrodeposition, it was washed using D.I. water, followed by drying 60 ˚C in air for 6 hours. Then, NiT_x/CP was obtained through anionization. The prepared samples were analyzed by X-ray diffraction (XRD). Supplementary Fig. 29b shows that the Ni/CP fully converted to NiT_x/CP after anionization.

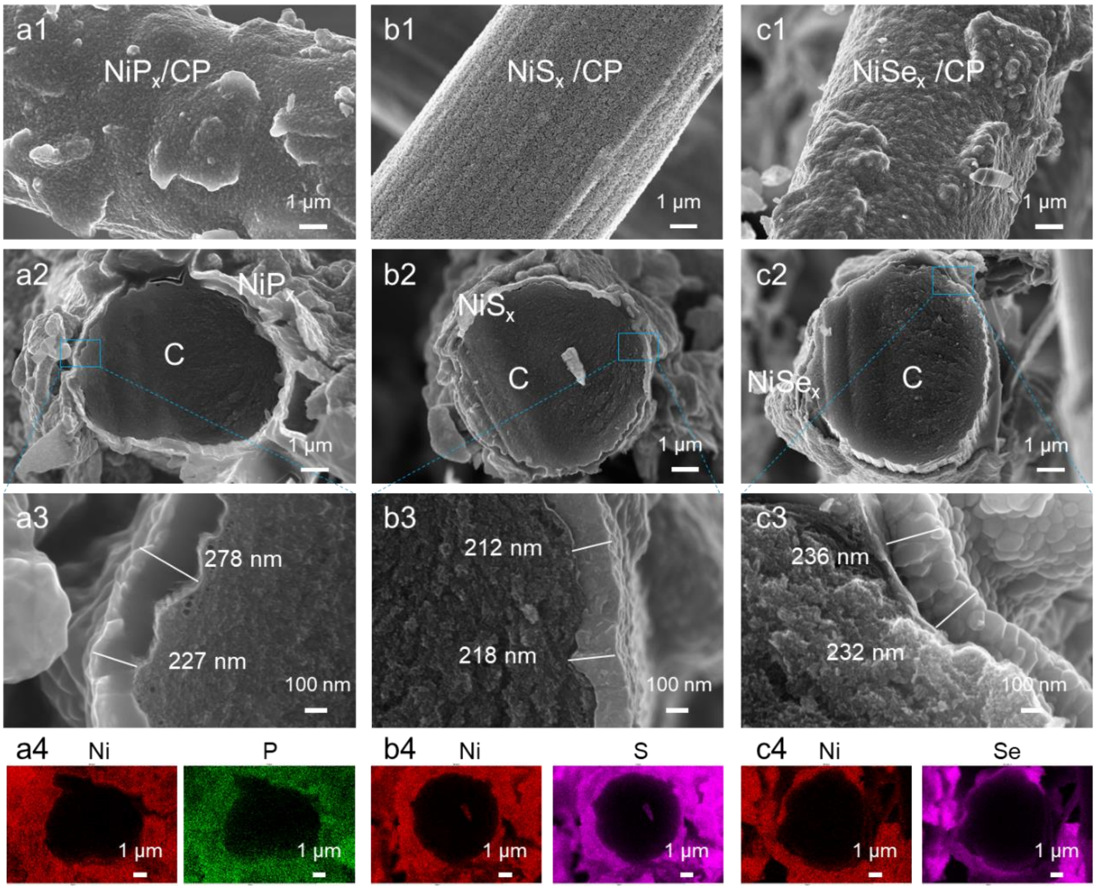

Supplementary Fig. 30 Morphology and compositional characterization of NiT_x/CP. SEM images (1), Cross-section SEM images (2-3) and elemental mapping (4) of NiP_x/CP (a), NiS_x/CP (b) and $NiSe_x$/CP (c).

Scanning electron microscopy (SEM) images (Supplementary Fig. 30 a1-a3) show that NiT_x compounds are successfully grown on carbon paper (CP). The thickness of NiT_x compounds on the CP surface is analyzed by cross-sectional SEM images and corresponding elemental mapping (Supplementary Fig. 30 a2-c4). The thickness of NiP_x, NiS_x and $NiSe_x$ is almost the same, around 210~230 nm.

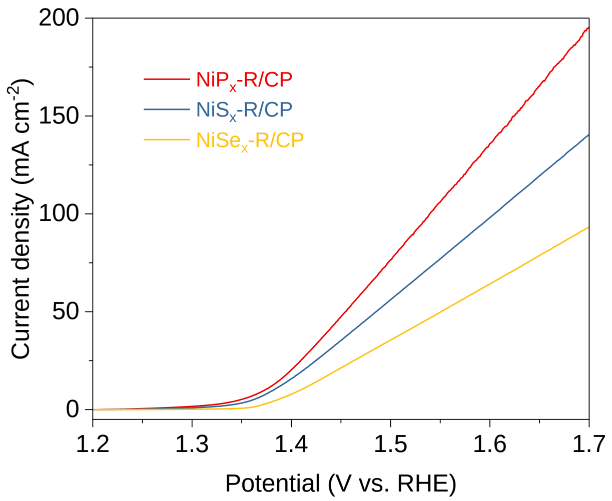

Supplementary Fig. 31 MOR performance of NiT_x-R/CP.
MOR polarization curves (without iR correction) of NiT_x-R/CP.

After electrochemical reconstruction in 1 M KOH, the MOR performance of the samples was tested in 1 M KOH containing 0.5 M methanol. After the cyclic voltametric activation, linear sweep voltammetry (LSV) at the scan rate of 5 mV s^{-1} without iR correction was performed to evaluate the MOR activity (Supplementary Fig. 31). Among these NiT_x-R/CP samples, NiP_x-R/CP exhibits the lowest onset potential and the best performance for MOR.

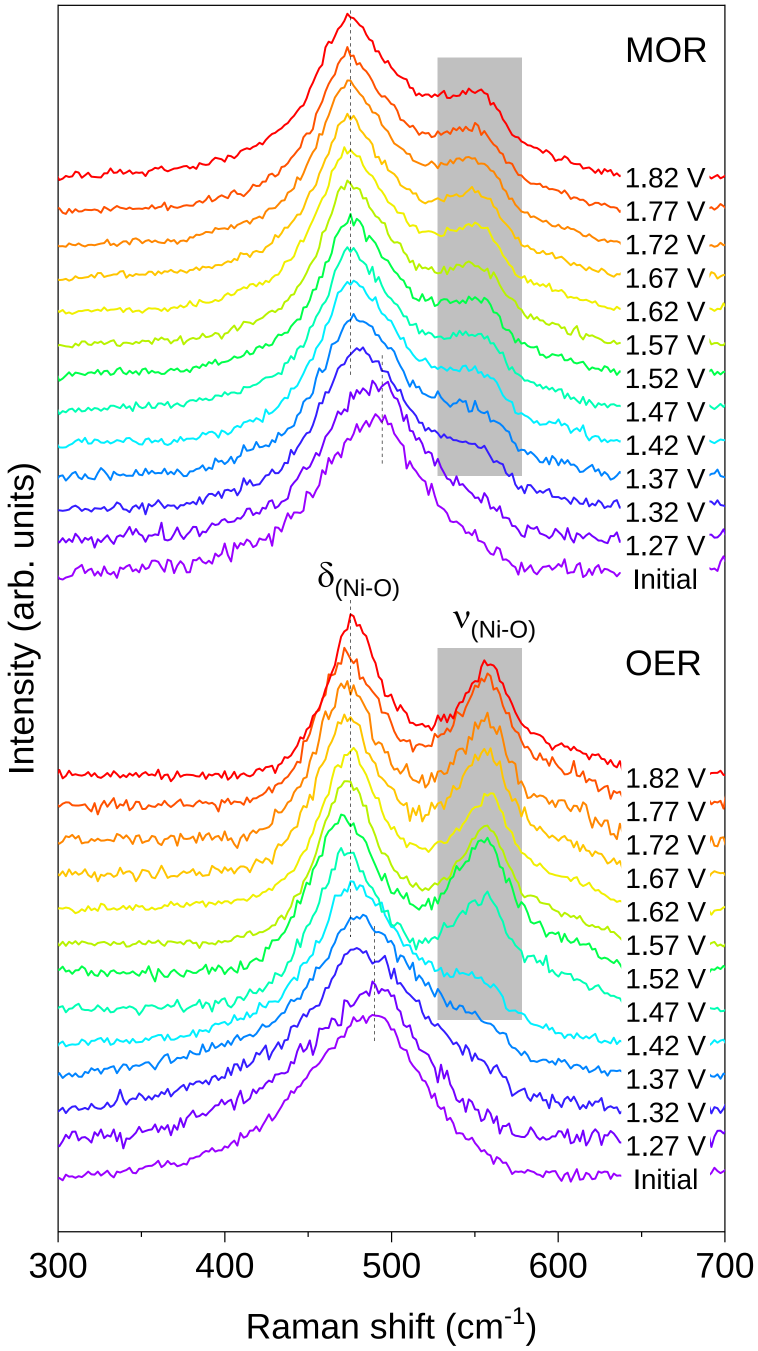

Supplementary Fig. 32 *In situ* Raman spectroscopy.
In situ Raman spectroscopy of NiP_x-R for OER (1 M KOH) and MOR (1 M KOH with 0.5 M methanol).

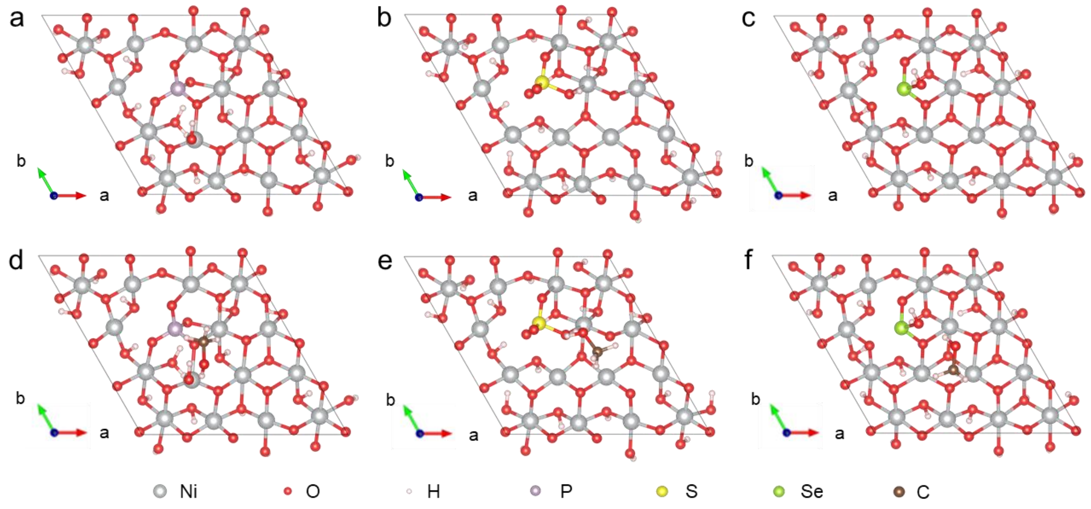

Supplementary Fig. S33 DFT calculations.

DFT optimized structures of the adsorbed intermediates on the NiOOH-TO_x surface. (a-c) OH* intermediates on the NiOOH-PO_x, NiOOH-SO_x, and NiOOH-SeO_x, respectively. (d-f) CH_3OH* adsorbed on the NiOOH-PO_x, NiOOH-SO_x, and NiOOH-SeO_x, respectively.

Supplementary Table 1 Structure parameters extracted from the Ni K-edge EXAFS fittings.

CN, coordination number; R, distance between absorber and backscatter atoms; σ^2, Debye-Waller factor to account for both thermal and structural disorders; ΔE, inner potential correction.

Catalyst	Scattering path	CN	R (Å)	σ^2	ΔE
NiP_x	Ni-P	0.73	2.314	0.003	-2.785
	Ni-Ni	1.60	2.604	0.010	
	Ni-O	2.58	2.053	0.006	
NiP_x-R	Ni-O	3.5	2.053	0.007	-3.995
	Ni-Ni	4.0	3.081	0.012	
	Ni-P	1.1	2.708	0.005	
	Ni-Ni(2)	2.9	2.750	0.020	
NiS_x	Ni-S	3.69	2.384	0.006	2.397
NiS_x-R	Ni-S	1.13	2.395	0.003	2.026
	Ni-O	2.15	2.055	0.005	
	Ni-Ni	0.78	3.065	0.005	
	Ni-S(2)	0.20	2.727	0.008	
	Ni-Ni(2)	1.46	2.819	0.030	
$NiSe_x$	Ni-Se	3.72	2.473	0.006	0.266
	Ni-O	1.15	2.070	0.017	
$NiSe_x$-R	Ni-O	4.2	2.049	0.007	-6.147
	Ni-Ni	5.1	3.083	0.010	
	Ni-Se	1.0	2.883	0.012	
	Ni-Ni(2)	0.9	2.819	0.012	

Supplementary Table 2 Comparison of the MOR activity reported in the previous works.

Catalysts	Activity	Condition	Reference
NiP_x-R	1.4 V (vs. RHE) at 400 mA cm^{-2}	1 M KOH and 0.5 M methanol	This work.
Ni_3S_2-CNFs	1.4 V (vs. RHE) at 100 mA cm^{-2}	1 M KOH and 1 M methanol	Nano Energy 80, 105530 (2021).
$Ni(OH)_2$/NF	1.36 V (vs. RHE) at 100 mA cm^{-2}	1 M KOH and 0.5 M methanol	Appl. Catal. B: Environ. 281, 119510 (2021).
Cu/NiCu NWs	867.1 mA mg_{metal}^{-1} at 1.55 V (vs. RHE)	1 M KOH and 1 M methanol	ACS Appl. Mater. Interfaces 9, 19843-19851 (2017).
h-NiSe/CNTs/CC	1.4 V (vs. RHE) at 100 mA cm^{-2}	1 M KOH and 1 M methanol	Adv. Funct. Mater. 31, 2008812 (2020).
V_O- NiO	85.3 mA cm^{-2} at 0.7 V vs. Ag/ AgCl	1 M KOH and 0.5 M methanol	Appl. Catal. B: Environ. 244, 1096-1102 (2019).
$Ni_{97}Bi$ metal aerogel	200 mA cm^{-2} at 0.65V vs. Ag/ AgCl	1 M KOH and 1 M methanol	Angew. Chem. Int. Ed. 59, 13891-13899 (2020).
$Ni_{0.75}Fe_{0.25}Se_2$	53.5 mA cm^{-2} at 1.55 V (vs. RHE)	1 M KOH and 1 M methanol	Small 17, e2006623 (2021).
Mn doped $Ni(OH)_2$	22 A g^{-1} at 1.55 V (vs. RHE)	1 M KOH and 0.5 M methanol	Nano Energy 55, 37-41 (2019).

Supplementary Table 3 Ni 3*d* band center (ε_d), O 2*p* band center (ε_p), total band center and energy difference (ΔE) between ε_d and ε_p of NiOOH-PO_x, NiOOH-SO_x, and NiOOH-SeO_x.

	ε_d (eV)	ε_p (eV)	Total band center (eV)	$\Delta E_{\varepsilon d - \varepsilon p}$ (eV)
NiOOH-SO_x	-1.636	-5.847	-1.594	4.211
NiOOH-PO_x	-1.684	-5.009	-1.133	3.325
NiOOH-SeO_x	-2.013	-7.239	-2.849	5.226
